# Tantalum–Hafnium: Optical Hydrogen Sensing Materials for High-Temperature Applications

**DOI:** 10.1021/acsami.5c09600

**Published:** 2025-07-18

**Authors:** Ilse van Ogtrop, Amy Navarathna, Herman Schreuders, Bernard Dam, Lars J. Bannenberg

**Affiliations:** † Faculty of Applied Sciences, 2860 Delft University of Technology, Mekelweg 15, 2629JB Delft, The Netherlands

**Keywords:** Optical Hydrogen Sensing, Metal hydrides, Thin Films, Tantalum, Hafnium, X-ray Diffraction, Neutron Reflectometry

## Abstract

Thin film metal hydride optical sensors, especially those made from tantalum, offer a large, hysteresis-free hydrogen sensing range, fast response times and great stability. However, due to the shift in tantalum’s hydrogen sensing ranges with rising temperatures, tantalum becomes inadequate for the detection of low hydrogen concentrations (<10^+3^ ppm) above 200 °C, making it unsuitable for high-temperature applications. We show that the properties of tantalum can be tailored by alloying tantalum with hafnium. Optical transmission measurements, ex situ and in situ X-ray diffraction and X-ray and neutron reflectometry are used to show that the introduction of Hf in Ta results in a solid solution with a stable structure with up to 21% Hf. Alloying Ta with Hf expands the unit cell, which alters the enthalpy of hydrogenation and shifts the sensing range to lower concentrations. Moreover, alloying Ta with Hf improves the sensitivity at low hydrogen concentrations (<10^+3^ ppm) and for temperatures exceeding 200 °C by about two times compared to pure Ta while preserving its large, hysteresis-free sensing range and excellent stability.

## Graphical abstract

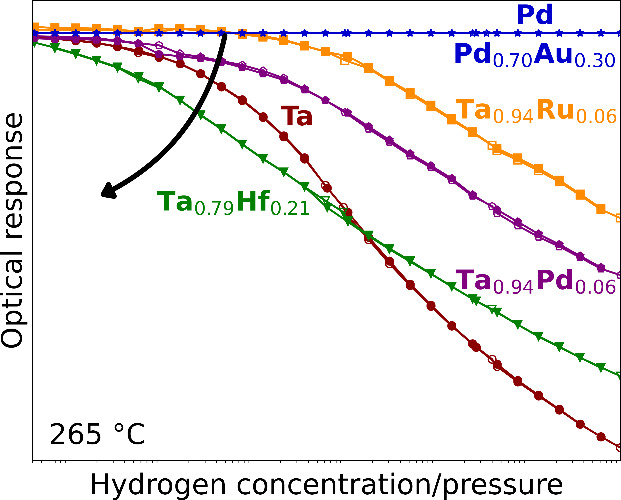

## 1 Introduction

Hydrogen is playing an increasingly important role in industrial applications and the transition to green energy.^1–4^ Hydrogen-air mixtures can be flammable or even explosive, making accurate and reliable sensing with hydrogen sensors essential for ensuring safety in environments where hydrogen is produced, stored, or used. The early detection of leaks is critical to ensure safety and as hydrogen is an indirect greenhouse gas.^5^ Additionally, hydrogen sensors are vital for monitoring of hydrogen concentrations during the operation of hydrogen fuel cells,^6^ CO_2_ conversion devices^7^ and various industrial processes.^8^

Current hydrogen sensors generally rely on electrochemical, catalytic or thermal conductive processes.^9,10^ They only operate over a small sensing range and are relatively large and costly. In contrast, optical hydrogen sensors can be made small and relatively inexpensive and have a high sensitivity over a large sensing range of up to 7 orders of magnitude in terms of partial hydrogen pressure or concentration. Most importantly, optical hydrogen sensors are intrinsically safe as they do not rely on electric currents near the sensing area, eliminating the possibility of the formation of sparks.^11,12^

The core component of an optical hydrogen sensor is an optical hydrogen sensing material. These materials, typically metal hydrides, absorb hydrogen when exposed to a hydrogen-containing environment, which causes a change in their optical properties. From the optical transmission or reflectivity of the material, one can determine the corresponding hydrogen concentration in the environment around the sensor.^13,14^ Ideal hydrogen sensing materials should possess a large sensing range, high sensitivity, and a fast, reversible, and hysteresis-free response. The response time is governed by the hydrogen dissociation, hydrogen diffusivity, and the absorbed amount of hydrogen. The sensing material will need a suitable structure to obtain a high hydrogen diffusivity and a catalytic capping layer can be used to catalyze the hydrogen dissociation.^15^ Lastly, to achieve a reversible and hysteresis-free optical response, the metal hydride must not undergo any phase transitions upon hydrogenation, and any plastic deformation needs to be avoided.^16,17^

The use of palladium and its alloys has been investigated extensively.^18–24^ Alloying palladium with, for example, gold is required to suppress the phase transition upon hydrogenation, which leads to an inherent hysteretic optical response. However, the alloying of palladium comes with the price of a severely reduced sensitivity of the sensor. Moreover, a residual hysteresis typically remains in thin films, due to the plastic deformation of the unit cell.^25^ As an alternative, tantalum and tantalum-based alloy thin-films show great promise due to their large sensing range of 7 orders of magnitude in partial hydrogen pressure, great sensitivity and hysteresis-free optical response.^16,19,26,27^ The large hysteresis-free sensing range results from the large hydrogen solubility range due to nanoconfinement, which also suppresses the phase transition seen in bulk tantalum. Tantalum’s elastic deformation upon hydrogenation eliminates any hysteresis caused by plastic deformation. Additionally, tantalum reaches fast response times due to the high hydrogen diffusivity, which is attributed to the body-centered cubic (BCC) structure.

An open challenge is to develop novel hydrogen sensing materials that can operate at high temperatures. There are many applications where the detection of hydrogen at high temperatures is required, such as the production of green steel (which uses hydrogen instead of coal),^28–30^ fertilizer production via the Haber–Bosch process,^31,32^ and hydrogen combustion engines.^33,34^ The current challenge is that the hydrogen sensing range of optical hydrogen sensing materials is temperature-dependent, shifting toward higher pressures as the temperature rises. This shift can be explained using the Van ’t Hoff law:

1 $$\ln\left(\frac{P_{eq}}{P_{0}}\right)=\frac{\Delta H}{RT}-\frac{\Delta S}{R}$$

where *P*_eq_ is the equilibrium hydrogen pressure, *P*_0_ the standard pressure (101.325 kPa), *R* the gas constant (8.314 JK mol^–1^), *T* the absolute temperature and Δ*H* and Δ*S* the enthalpy and entropy of the hydrogenation reaction, respectively.^12^ Both Δ*H* and Δ*S* have negative values for metal hydride hydrogen sensing materials.^35^ As a result, the equilibrium pressure needed to reach a certain hydrogenation level increases with the temperature. This effectively shifts the entire sensing range to higher pressures. The commonly used palladium and palladium–gold are completely unsuitable for the detection of hydrogen at high temperatures as the optical response diminishes completely at elevated temperatures (*T* > 150 °C).^36^ In comparison, tantalum provides an excellent optical response at moderate hydrogen concentrations (0.1 < *c*_H_2__ < 10%). However, the optical response at lower concentrations (1.0 < *c*_H_2__ < 10^+3^ ppm) is weak at temperatures over 200 °C, making tantalum sensors inadequate for high-temperature applications.

The properties of tantalum may be tailored by alloying. Both palladium and ruthenium have been successfully used as alloyants.^26,27^ In that case, the unit cell contracts, due to the smaller atomic volume of Pd (0.0142 nm^3^) and Ru (0.0136 nm^3^) compared to Ta (0.0180 nm^3^).^37^ The compressive strain resulting from the smaller unit cell reduces the absolute enthalpy of hydrogenation.^26^ In turn, the amount of hydrogen absorbed at a certain pressure decreases, which shifts the sensing range of tantalum toward higher pressures.

Taking a similar, yet opposite approach, an opportunity to shift the sensing range of tantalum to lower hydrogen concentrations would be to expand the Ta unit cell and increase the hydrogenation enthalpy by alloying it with an element larger than Ta.^38^ Apart from being larger in size, this element should form a solid solution with Ta over a reasonable alloyant concentration to ensure structural stability of the material and maintain the excellent properties of tantalum as a sensing material. In this light, hafnium is a promising candidate: it has a substantially larger atomic volume (0.0223 nm^3^) compared to tantalum and it is able to form a solid solution with Ta up to ∼25% in bulk.^37,39^

The purpose of the present paper is to investigate the suitability of Ta–Hf as an optical sensing material. Specifically, the focus is on whether alloying tantalum with hafnium increases the sensitivity for low partial hydrogen pressures/concentrations at high temperatures. Using X-ray diffraction, we find that Ta_1–*y*_Hf_*y*_ thin films with 0.00 ≤ *y* ≤ 0.21 form a solid solution with a BCC structure. Optical measurements confirm a shift in the sensing range to lower pressures while maintaining the hysteresis-free response over 7 orders of magnitude in terms of hydrogen pressure. Due to the shift in sensing range, the Ta–Hf alloys have reached a sufficient hydrogen detection at temperatures of 210–270 °C for low hydrogen concentrations.

## 2 Experimental Method

### 2.1 Sample Deposition

The Ta_*y*–1_Hf_*y*_ thin films were prepared by magnetron sputtering. Each sample consists of a 4 nm titanium adhesion layer that promotes the growth of the Ta alloy in the desired BCC structure,^26,27^ a 40 nm Ta_*y*–1_Hf_*y*_ sensing layer, and a 10 nm Pd_0.6_Au_0.4_ capping layer. The capping layer is used to prevent the oxidation of the sensing layer and to catalyze the hydrogen dissociation and recombination reaction.

All thin films for the X-ray diffraction (XRD), X-ray reflection (XRR) and hydrogenography measurements were deposited on 10 mm × 10 mm quartz substrates, with a thickness of 0.5 mm and surface roughness of <0.4 nm (MaTeck GmbH, Jülich, Germany). For the neutron reflectometry measurements, circular fused quartz substrates with a diameter of 3 in., a thickness of 5.0 mm, a surface roughness of <0.5 nm and a flatness of 2 lambda over 85% CA Central (Coresix Precision Glass, Inc., VA, U.S.A.) were used. The metallic layers were deposited in 0.3 Pa of Ar via direct current magnetron sputtering in an ultrahigh vacuum chamber (AJA Instruments) with a base pressure between 10^–7^ and 10^–9^ hPa. The samples were rotated during the deposition to enhance the homogeneity of the layers.

Typically, deposition rates of 0.14 nm s^–1^ (130 W DC) for Ta, 0.15 nm s^–1^ (130 W DC) for Hf, 0.03 nm s^–1^ (100 W DC) for Ti, 0.13 nm s^–1^ (50 W DC) for Pd and 0.11 nm s^–1^ (25 W DC) for Au were used. The deposition rates of Ta and Hf were altered specifically for each composition, where *y* in Ta_1–*y*_Hf_*y*_ ranges from 0.00 to 0.80. The specific sputter conditions for each composition can be found in Table S1. In addition, an optical reference sample consisting of a 4 nm Ti adhesion layer, a 40 nm Ta_0.50_Pd_0.50_ layer and a 10 nm Pd_0.60_Au_0.40_ capping layer was made. The 40 nm Ta_0.50_Pd_0.50_ layer does not hydrogenate and, thus, does not change its optical transmission upon exposure to hydrogen. This makes it possible to measure the optical properties of the Pd_0.6_Au_0.4_ capping layer while retaining a similar layer structure to that of the Ta_1–*y*_Hf_*y*_ samples. The thicknesses of each layer are confirmed by XRR measurements (see below for experimental details), which show that the maximum deviation of the desired thickness of the sensing layer is 2.6 nm (6.6%). Additionally, it shows that the roughness is typically <1.0 nm and that the density of the film decreases linearly with increasing Hf concentration (Figure S1 and Table S2). The composition of the sensing layer is assed with energy dispersive X-ray spectroscopy (EDS). The results of the EDS analysis of the Ta_0.79_Hf_0.21_ thin film are presented in Table S3 and show that there is 20.7% of Hf based on the atom% of Ta and Hf. Figure S2 shows a scanning electron microscopy (SEM) image of the material.

### 2.2 Structural Measurements

The structure and thickness of all samples was assessed with XRD and XRR. Out-of-plane ex situ and in situ XRD and XRR experiments were performed with a Bruker D8 Discover diffractometer (Cu Kα, λ = 0.1542 nm) equipped with a LYNXEYE XE detector operating in 0D mode (Bruker AXS, Karlsruhe, Germany). The ex situ XRD measurements were performed with a Göbel mirror, a 0.6 mm fixed exit slit on the primary side, and two 0.6 mm slits on the secondary side. The ex situ XRR measurements were performed with a Göbel mirror, a 0.1 mm fixed exit slit, and two 0.1 mm slits on the secondary side.

In-plane ex situ XRD measurements were used to further assess the structure by identifying additional diffraction peaks that belong to other crystal planes in the textured thin films. The in-plane measurements were performed with the detector operating in 1D mode, the motorized slit set to a fixed sample illumination of 6 mm and a PSD opening of 2.86°. An Eulerian cradle sample stage was used to adjust the in-plane angle, *χ,* from 0 to 80° with increments of 5°.

The in situ XRD and XRR measurements were performed with a Göbel mirror, a 0.2 mm fixed exit slit on the primary side, two 0.2 mm slits on the secondary side, and the detector operating in 0D mode. The measurements were performed in an Anton Paar XRK900 Reactor chamber with a base pressure of 5 × 10^–4^ mbar (Anton Paar GmbH, Graz, Austria). A solenoid inlet valve (MKS Inst. 0248AC-10000SV) was connected to a pressure controller (MKS Inst., Inc., type 250 controller, Andover, MA, U.S.A.) to regulate the pressure of the reactor chamber. The outlet of the chamber was connected to a parallel arrangement of a mass flow controller (Brooks Instruments 150 sccm, Hatfield, PA, U.S.A.) and solenoid outlet valve (MKS Inst., Inc., 0248AC-10000SV, Andover, MA, U.S.A.) which was directed by a power supply unit (Delta Elektronica ES030–5 Power Supply, Zierikzee, The Netherlands) and onward to a vacuum pump (Adixen Drytel 1025, Pfeiffer Vacuum GmbH, Asslar, Germany). A National Instruments LabVIEW code was used to control the pressure-control unit, outlet valve (via the power supply unit), and the mass flow controller.

The in situ XRD and XRR measurements were performed with decreasing pressure steps using a 4% H_2_ in He gas mixture (Δ*c*_H_2__/*c*_H_2__ < 2%, Linde Gas Benelux BV, Dieren, The Netherlands). Generally, a gas flow of 10 sccm was used, and between the pressure steps with a larger difference, a 100 sccm gas flow was used. The in situ XRD and XRR measurements were performed at multiple temperatures, where the temperature of the system is controlled with an Antor Paar TCU 700 control unit.

The amount of hydrogen absorbed by the sensing layer was studied by neutron reflectometry. The time-of-flight neutron reflectometry measurements were carried out using the ROG neutron reflectometer, which is connected to the 2.3 MW Hoger Onderwijs Reactor (HOR) of the Delft University of Technology, Delft, The Netherlands. The double disk chopper was set to a frequency of 13.2 Hz with an interdisc distance of 0.280 m to achieve a wavelength resolution of Δλ/λ≈ 2.5%. This resulted in a spectrum of 0.4 < λ < 1.2 nm. A series of measurements with incident angles of 8, 16, 24, and 32 mrad, respectively, were made and combined together to obtain a *Q* – range of 0.1 < *Q* < 1.0 nm^–1^ with the given wavelength range. The first and second slits were adjusted for each incident angle, resulting in a footprint of 40 × 60/80 mm^2^ (umbra/penumbra) and a resolution of Δ*Q*/*Q* ≈ 4.2%. A position sensitivity detector (PSD) was used to detect the reflected neutrons.^40^ The Ta_*y*–1_Hf_*y*_ thin films were hydrogenated at a temperature of 25 °C and 273 ± 5 °C. The partial H_2_ pressure is varied by stepwise adjusting the absolute pressure of 0.1% and 4.0% H_2_ in Ar gas (Δ*c*_H_2__/*c*_H_2__ < 2%, Linde Gas Benelux BV, Dieren, The Netherlands). Generally, a gas flow of 20 sccm was used, and between the pressure steps with a large pressure difference, a 100 sccm gas flow was used.

The XRR and neutron reflectometry data were fitted using GenX3.^41^ A three-layer model was used to estimate the thickness, roughness, and scattering length density (SLD) of each layer. The thickness of the Ti and Pd_0.6_Au_0.4_ layers was kept constant in the analysis as our main interest is the thickness of the Ta_1–*y*_Hf_*y*_ layer. The Pd_0.6_Au_0.4_ layer can also absorb H_2_ resulting in an expansion. This leads to a small overestimation of the expansion of the Ta_1–*y*_Hf_*y*_ layer. The hydrogen concentration, *x* in Ta_1–*y*_Hf_*y*_H_*x*_, can be calculated using the found thicknesses and SLDs of the layers with the following equation

2 $$x=\left(\frac{SLD_{Ta_{1-y}Hf_{y}H_{x}}}{SLD_{Ta_{1-y}Hf_{y}}}\frac{d_{Ta_{1-y}Hf_{y}H_{x}}}{d_{Ta_{1-y}Hf_{y}}}-1\right)\frac{\left(1-y\right)b_{Ta}+yb_{Hf}}{b_{H}}$$

where SLD = ∑_*i* = 1_^*N*^ *b*_*i*_ *N*_*i*_ is the SLD of the layer; *b*_Ta_ = 6.91 fm, *b*_Hf_ = 7.77 fm and *b*_H_ = −3.739 are the scattering lengths of Ta, Hf and H, respectively;^42^ and *N*_*i*_ the number of atoms *i* per volume unit.^26^ A full derivation of eq 2 is given in the Supporting Information (SI) of Bannenberg et al. (2024).^43^

The morphology of the surface is assessed with atomic force microscopy (AFM). The AFM measurements were performed on a Bruker’s Dimension Icon AFM run in ScanAsyst mode. For each thin film, 500 nm and 2 μm scans were recorded. The AFM measurements were performed after the thin films were exposed to hydrogen and 270 °C. The results can be found in Figure S3 and reveal no in-plane features and a relative smooth surface.

### 2.3 Optical Measurements

Hydrogenography was used to investigate the optical transmission of the samples.^44^ An Imaging Source DFK 23UM021 1/3 in. Aptina CMOS MT9M021 1280 × 960 pixel color camera with a Fujinon HF9HA-1S lens was used to record the transmission. The lamp (powered by Delta Elektronica ES030–10 Power Supply, Zierikzee, The Netherlands) consists of 5 Philips MR16 MASTER LEDs (10/50 W) with a color temperature of 4000 K. The optical transmission was separately measured in the red, green, and blue channels. The spectral sensitivity of the three color channels and the intensity of the LEDs can be found in the SI of Bannenberg et al. (2023).^26^ An infrared filter is used to reduce the contribution of infrared light in each channel. The optical transmission from the green channel is presented unless otherwise specified. The transmission was averaged over an area of approximately 80 mm^2^ for each sample. The measurements were corrected with the optical reference sample to compensate for the influence of the Pd_0.6_Au_0.4_ capping layer on the transmission, as well as fluctuations of the light source. The partial H_2_ pressure was varied from 10^–1^ to 10^+6^ Pa by using 0.1%, 4.0%, and 100% H_2_ in Ar gas mixtures (Δ*c*_H_2__/*c*_H_2__ < 2%, Linde Gas Benelux BV, Dieren, The Netherlands) and by varying the absolute pressure inside the chamber. A gas flow of 10 or 20 sccm was used for increasing pressure steps, and a gas flow of 200 sccm was used for decreasing pressure steps. All optical measurements were conducted with a relative humidity of 0%.

The wavelength dependence of the optical response was studied to determine which wavelength of light produces the highest optical contrast. A halogen and deuterium light source (Avantes Avalight-DH-S-BAL, Apeldoorn, The Netherlands) and an Ocean Optics Maya 2000 Pro spectrometer with an acquisition frequency of 2.5 Hz were used to measure the transmission spectrum at a specific H_2_ concentration. Flow controllers (Brooks Instrument GF40 Thermal mass flow controller and meter, Hatfield, USA) were used to regulate the incoming flow and, if needed, mix H_2_ gas with Ar for lower concentrations. The spectrum was recorded once every second for ± 10 min, and the average of all the recorded spectra was subsequently used.

## 3 Results

### 3.1 Structural Properties

#### 3.1.1 Ex Situ XRD

For an optical hydrogen sensor, a single phase material is preferred as this likely promotes the long-term stability and reduces the chance of hysteresis. In addition, we aim for a BCC structure since these are known to allow fast diffusion of hydrogen and thus allow a quick response of the sensor.^27^ XRD shows that all Ta_1–*y*_Hf_*y*_ thin films are strongly textured with the (110) BCC Ta and (111) face-centered cubic (FCC) PdAu planes in the out-of-plane direction (See Figure 1A, the full diffraction patterns can be found in Figure S4). Compositions with higher Hf-concentrations than *y* = 0.30 have been deemed uninteresting for hydrogen sensing applications, and all relevant results of these compositions can be found in the SI (Text 1).

**Figure 1:**
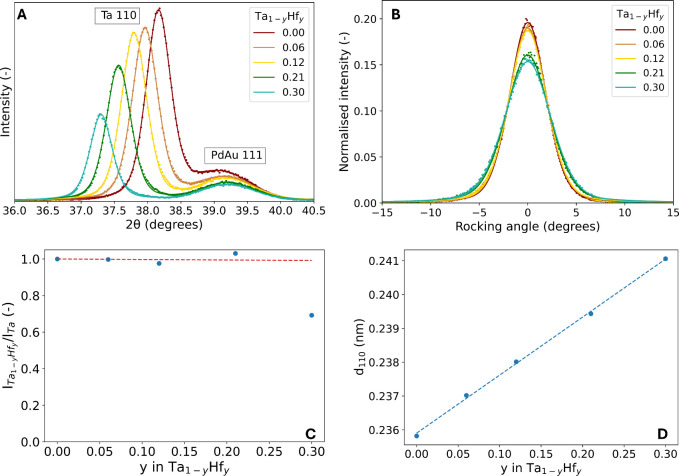
Ex situ XRD results of the as-prepared 40 nm Ta_1–*y*_Hf_*y*_ thin films with a 4 nm Ti adhesion layer and a 10 nm Pd_0.6_Au_0.4_ capping layer. A) Diffraction patterns of the Ta_1–*y*_Hf_*y*_ thin films. The continuous lines represent fits of two pseudo-Voight functions to the experimental data presented by the dots. B) Rocking curves of the Ta_1–*y*_Hf_*y*_ thin films around the Ta (110) peak normalized with the integrated intensity of the experimental data and centered around zero. The continuous lines represent the fits of a pseudo-Voight function to the normalized data represented by the dots. C) Hf concentration dependence of the peak intensity of the Ta (110) peak for Hf concentration of 0.00 ≤ *y* ≤ 0.30. The peak intensity is computed by multiplying the integrated intensity of the fitted Ta (110) peak with the square of the fwhm of the rocking curves and scaled to the intensity of the Ta sample. The dashed red line represents the theoretical expected Hf-content dependence of the intensity based on the difference in *Z* between Ta and Hf (see SI Text 1). D) The Hf concentration dependence of the *d*_110_-spacing in Ta_1–*y*_Hf_*y*_. The blue fitted line is based on the blue markers of the *d*_110_-spacing for 0.00 ≤ *y* ≤ 0.30.

The absence of any additional diffraction peaks suggests that the Ta_*y*–1_Hf_*y*_ thin films form a solid solution for 0.00 ≤ *y* ≤ 0.30. However, we observe a significantly lower intensity (total area of the peak) of the Ta (110) BCC diffraction peak of *y* = 0.30 compared to 0.00 ≤ *y* ≤ 0.21. To compare the intensities of the diffraction peaks in more depth, the rocking curves shown in Figure 1B also need to be considered. The full width at half maximum (fwhm) of the rocking curves increases with higher Hf contents, which indicates that the mosaic spread increases. Figure 1C shows the intensity of the 0.00 ≤ *y* ≤ 0.30 diffraction peaks, taking into account the fwhm of the rocking curves and the total area of the diffraction peaks. Theoretically, one expects that the intensity of the diffraction peaks is almost independent of Hf substitution (SI Text 2). For 0.00 ≤ *y* ≤ 0.21, the experimental data align with the theoretical intensity. This suggests that no secondary phases are present. However, there is a clear deviation between the experimental data and the theoretical behavior for *y* = 0.30. In this case, a decrease in the intensity could be attributed to the formation of an additional phase. In-plane XRD measurements reveal only reflections that can be attributed to a BCC crystalline phase (see Figure S5), so the additional phase of *y* = 0.30 is likely to be amorphous or microcrystalline in nature.

Significantly, the shift of the (110) BCC diffraction peaks to lower 2θ angles for 0.00 ≤ *y* ≤ 0.30, indicates an increase in the unit cell lattice parameter with increasing Hf concentrations. The increase in the unit cell lattice parameter is highlighted by Figure 1D, which illustrates a linear relationship between the *d*_110_-spacing and the Hf concentration for 0.00 ≤ *y* ≤ 0.30 and is consistent with the formation of a solid solution. The expansion is substantial, amounting up to 2.2% for *y* = 0.30, and thus potentially results in a large tunability of the equilibrium hydrogen pressures of the material. The expansion of the *d*_110_-spacing highlights the achievement of a primary goal in this research: obtaining a larger unit cell through alloying Ta with Hf.

#### 3.1.2 In Situ XRD and XRR

Phase transitions should be avoided in hydrogen sensing materials as (first-order) phase transitions lead to hysteresis in the optical response, longer response times and a compromised stability over repeated cycling.^19,26,45,46^ Additionally, the structure should not plastically deform upon hydrogenation, as this may act as another source of hysteresis. We study the presence of a phase transition and plastic deformation effects by in situ XRD and XRR. The unloaded state refers to a measurement in air after the thin film is exposed to hydrogen. The diffraction peaks of this state have slightly shifted to higher angle compared to the measurements in Figure 1, indicating a more compact structure due to the settlement of the microstructure after the first exposure to hydrogen. This is a phenomenon commonly observed for magnetron sputtered metal hydride thin films.^15,26^

Within a limited compositional range (0.00 ≤ *y* ≤ 0.12), in situ XRD does not show any indication for a phase transition on exposure to hydrogen or the presence of a secondary phase (Figures 2A, 2B and S6). No additional diffraction peaks appear, and despite a slight change in the width of the diffraction peak, the intensity (total peak area) and peak shape of the diffraction peaks remain the same. In contrast, the intensity and total peak area of the *y* = 0.21 diffraction peaks decreases upon hydrogenation (Figure 2C). In situ rocking curves (see Figure S7) show that the fwhm of the rocking curve significantly increases upon hydrogenation, which indicates increased mosaic spread, increased (micro)­strain, or loss of crystalline order due to defects or disorders. The larger fwhm explains the loss of intensity of the corresponding diffraction peak in the textured thin-film as the distribution of orientations of the planes increases and less are oriented in the out-of-plane direction upon hydrogenation. This change in orientation is reversible, shown by the intensity of the unloaded state which is similar to the as-prepared state. Overall, the loss in intensity probably will not negatively affect the optical properties of *y* = 0.21, as it is likely related to the orientation of the crystallites and is reversible.

**Figure 2:**
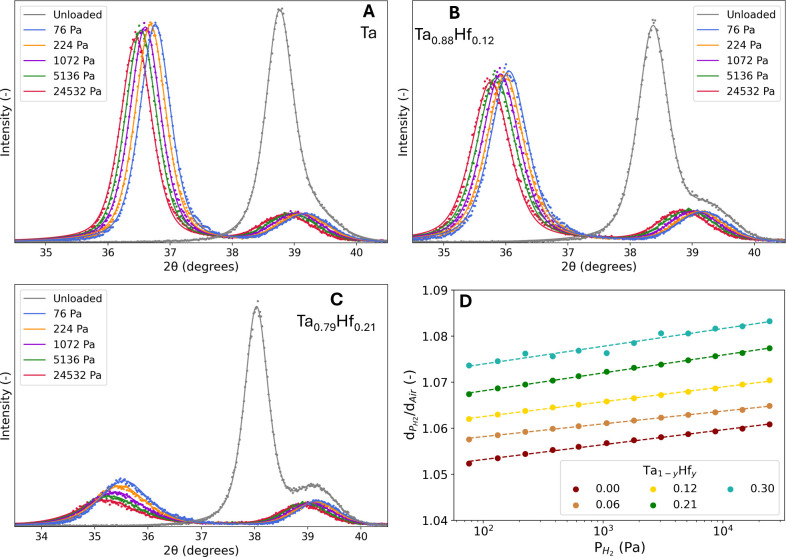
In situ XRD results of a 40 nm A) Ta, B) Ta_0.88_Hf_0.12_ and C) Ta_0.79_Hf_0.21_ thin film with a 4 nm Ti adhesion layer and a 10 nm Pd_0.6_Au_0.4_ capping layer at 25 °C. The continuous lines represent the fits of two pseudo-Voight functions to the experimental data. Diffraction patterns are measured at the indicated partial H_2_ pressures and for decreasing pressure steps. D) The dependence of the expansion of the *d*_110_-spacing relative to the unloaded state in air, *d*_Air_, on the partial hydrogen pressure. The dashed lines represent linear fits to the experimental data presented by the dots.

Furthermore, the diffraction peaks of the sensing layer shift smoothly to lower angles when increasing the partial hydrogen pressure, revealed by a lattice expansion of the sensing layer by up to 6% and 7% in terms of *d*_110_-spacing at 10^+4^ Pa for *y* = 0.06 and *y* = 0.21, respectively (see Figure 2D). Evidently, Hf-substitution increases the amount of relative lattice expansion compared to pure Ta. The gradual expansion is underlined by the complementary in situ XRR results (Figure 3A and 3B), which show a gradual expansion of the layer thickness with increasing partial hydrogen pressure in Figure 3C. The XRR measurements indicate that the volumetric expansion increases with the Hf concentration, similar to the *d*_110_-spacing.

**Figure 3:**
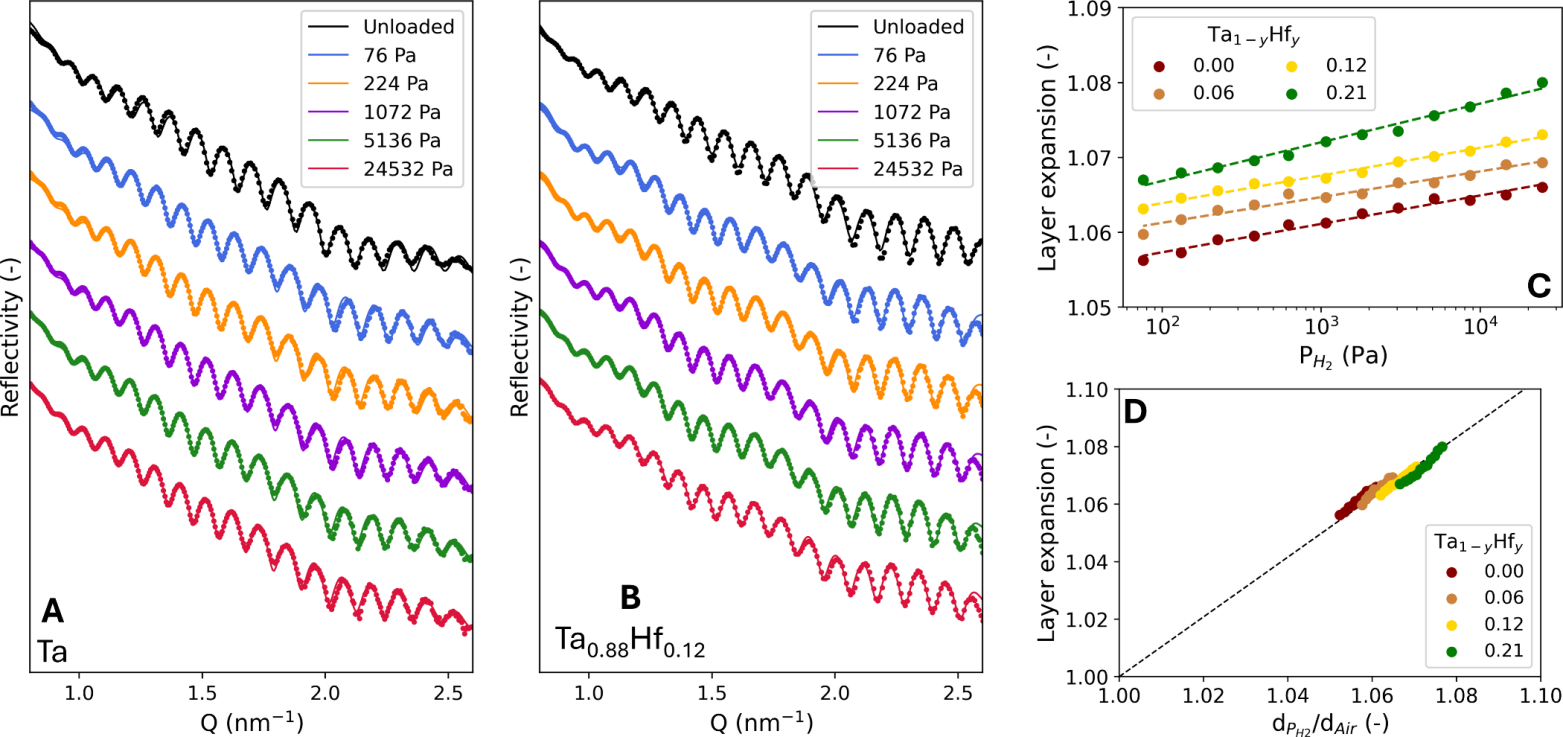
In situ XRR measurements of a 40 nm A) Ta and B) Ta_0.88_Hf_0.12_ thin film with a 4 nm Ti adhesion layer and a 10 nm Pd_0.6_Au_0.4_ capping layer at 25 °C. The continuous lines represent the fits of a model to the experimental data presented by the dots. The thicknesses and densities of the capping and adhesion layers are kept constant for each measurement. C) The dependence of the Ta_1–*y*_Hf_*y*_ layer expansion on the partial hydrogen pressure. The dashed lines represent fits to the experimental data presented by the dots. D) Relation between the d_110_-spacing and layer thickness expansion. The black dashed line serves as a guide to the eye.

The relation between the *d*-spacing and layer thickness can be used to study the nature of the expansion of the sensing material. In bulk Ta, the expansion is accommodated in all directions, making the relationship between the volume of a cubic unit cell and the *d*-spacing *V*∝*d*_*hkl*_^3^. However, in the case of clamped Ta thin films textured in the 110 direction, the expansion is only realized in the out-of-plane direction, making *V*∝*d*_*hkl*_.^26,43^ The differences in behavior between bulk Ta and a clamped Ta thin film can be attributed to the nanoconfinement of the thin film. With the expansion in only the out-of-plane direction, the cubic unit cell will deform and become slightly tetragonal upon hydrogenation. For the Ta_1–*y*_Hf_*y*_ thin films with 0.00 ≤ *y* ≤ 0.21, *V*∝*d*_110_ is applicable as is indicated by the black dashed line in Figure 3D. The deformation is also elastic in nature, which prevents hysteresis in the optical response that would result from plastic deformation.

The in situ XRD and XRR measurements were repeated at 270 °C to investigate the structural stability of the thin films at high temperatures. All results are provided in Figure S8. In this case, the XRD patterns of the unloaded state show well-defined peaks that are shifted to slightly smaller angles due to a thermal expansion of approximately 0.2% in terms of *d*-spacing. We do not find a significant change in the peak shape, and nor were there any additional diffraction peaks found, indicating that also at 270 °C the 0.00 ≤ *y* ≤ 0.21 alloys remain phase pure. Most importantly, the SLD profiles of the complementary XRR show well-defined steps at the interface of each layer. This indicates that there is no layer mixing or alloy formation, suggesting that the layers remain intact when the temperature is increased. Overall, the thin films are structurally stable at 270 °C.

#### 3.1.3 Neutron Reflectometry

The amount of hydrogen absorbed is a key property of the sensing material. The optical properties of the sensing material change upon hydrogenation as the material absorbs hydrogen. To determine the hydrogen content, in situ neutron reflectometry measurements are used (see Section 2.2 and eq 2). The results can be used to determine the pressure composition isotherms (PCIs): the relation between the partial hydrogen pressure and the hydrogen content *x* for a given material and temperature. The PCIs are presented in Figure 4 and the corresponding raw data, fits, and SLD profiles can be found in Figures S9 and S10.

**Figure 4:**
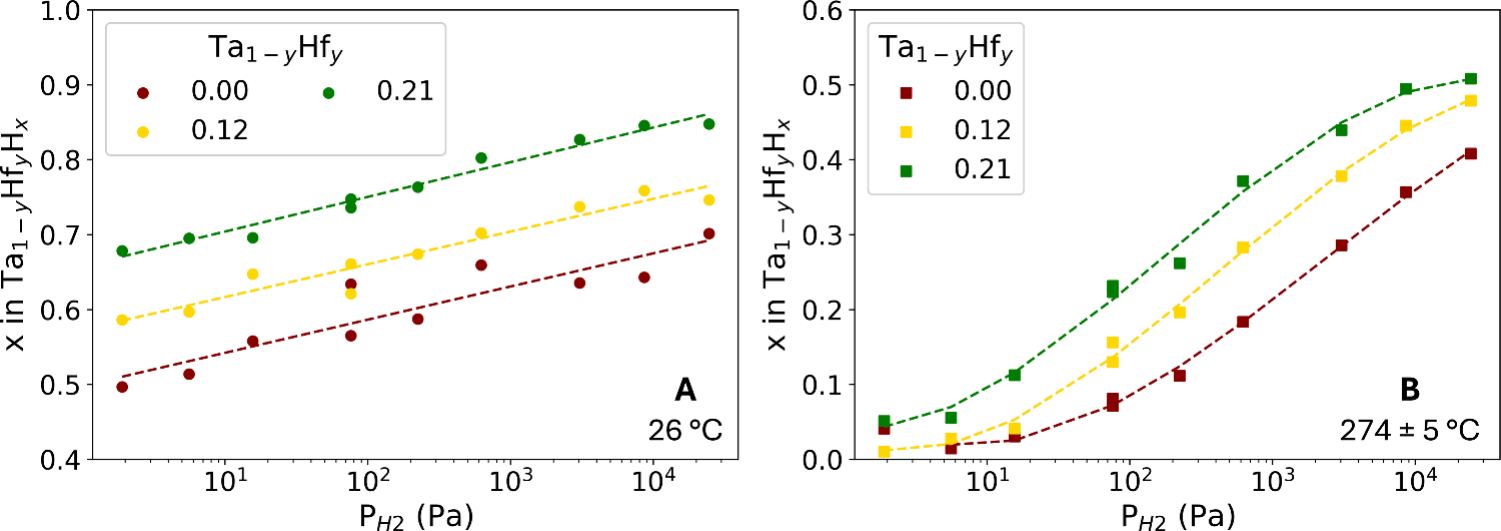
In situ neutron reflectometry results of a 40 nm Ta_1–*y*_Hf_*y*_ thin film with a 4 nm Ti adhesion layer and a 10 nm Pd_0.6_Au_0.4_ capping layer. The data with corresponding fits and SLDs can be found in Figures S9 and S10. The dependence of the hydrogen pressure on the hydrogen content of the nm Ta_1–*y*_Hf_*y*_ layer at A) 26 °C and B) 274 ± 5 °C. The dotted lines represent the fits of a linear (A) or third-degree polynomial (B) function to the experimental data. The results of the Ta thin film at 25 °C are adapted from ref 16 with permission from the authors. Available under a CC-BY 4.0 license. Copyright 2023 Bannenberg et al.

Neutron reflectometry shows that the Hf substitution increases the amount of hydrogen absorbed at any given partial hydrogen pressure both at room temperature (Figure 4A) and at 274 ± 5 °C (Figure 4B). The increased amount of absorbed hydrogen is consistent with the larger layer expansion and relative *d*-spacing upon hydrogen exposure found by in situ XRD and XRR (Figure 2 and Figure 3). Furthermore, with a higher amount of Hf, hydrogen is starting to be absorbed at lower pressures at higher temperatures. Specifically at 274 ± 5 °C, it is apparent that the PCIs are shifted horizontally to lower pressures with an increasing amount of Hf, showing that the hydrogen sensing range is shifted to lower pressures. To establish whether we achieve sufficient sensitivity to detect low partial hydrogen pressures at high temperatures, we determined the optical properties of the materials in the following section.

### 3.2 Optical Properties

#### 3.2.1 Optical Response and Sensing Range

The optical response of the sensing materials is the most important factor in the overall performance of the material as an optical hydrogen sensor. It is assessed with hydrogenography, which is based on measuring the optical transmission of the thin films when exposed to H_2_. The change in the optical transmission *T* relative to the transmission of the unloaded state *T*_0_ is measured, while the partial hydrogen pressure is increased and decreased in a series of steps. The optical transmission of the Ta_1–*y*_Hf_*y*_ thin films over three different pressure ranges is presented in Figure 5.

**Figure 5:**
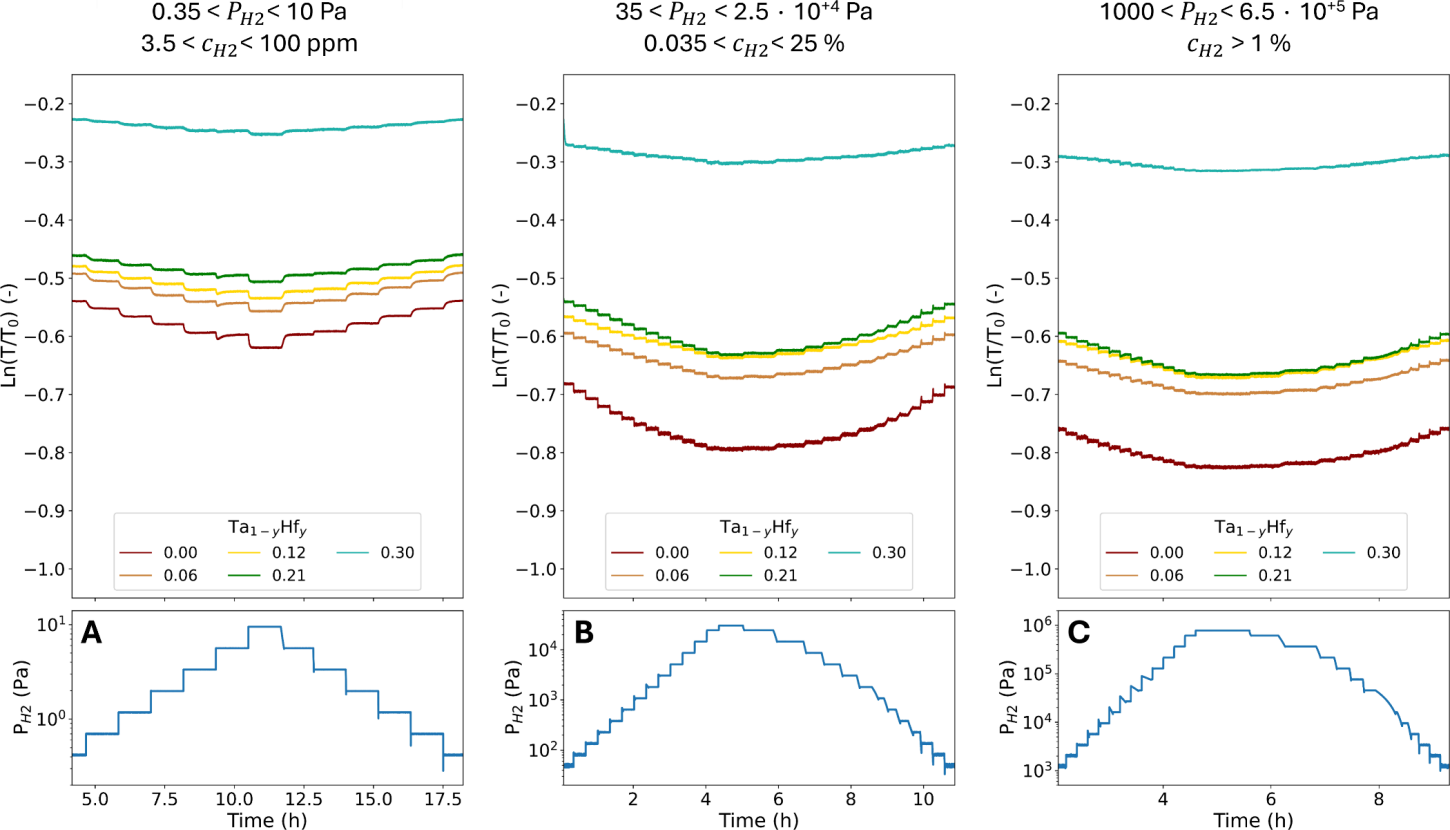
Changes of the optical transmission *T* relative to the transmission of the unloaded state *T*_0_ of the 40 nm Ta_1–*y*_Hf_*y*_ thin films with a 4 nm Ti adhesion layer and a 10 nm Pd_0.6_Au_0.4_ capping layer. The contribution of the capping layer was subtracted by subtracting the optical response of the Ta_0.5_Au_0.5_ reference thin film with the same adhesion and capping layer. The thin films were exposed at 25 °C to various increasing and decreasing pressure steps of A) 1.5 × 10^–1^ < *P*_H_2__ < 1.0 × 10^+1^ Pa, B) 3.5 × 10^+1^ < *P*_H_2__ < 2.5 × 10^+4^ Pa, and C) 1.0 × 10^+3^ < *P*_H_2__ < 6.5 × 10^+5^ Pa. The indicated hydrogen concentration ranges are determined for an environment with a total pressure of 10^+5^ Pa.

The 0.00 ≤ *y* ≤ 0.30 thin films show a decrease in the optical transmission with increasing pressure. The transmission steps are well-defined and stabile at any partial hydrogen pressure. The change in transmission is monotone with increasing partial hydrogen pressure. Additionally, the optical transmission of the thin films is equal for increasing and decreasing steps of partial hydrogen pressure making the optical response free of any hysteresis.

The optical response of the sensing layers can be summarized into pressure-transmission isotherms (PTIs). Similar to a PCI, a PTI represents the partial hydrogen pressure dependence of the *optical transmission* for a given material and temperature. Figure 6 presents the PTIs at 26 and 267 °C for 0.00 ≤ *y* ≤ 0.30. The PTIs show that the decreasing and increasing pressure data points overlap, confirming the hysteresis-free optical response over a wide sensing range at both low and high temperatures. Additionally, Figure 6B shows that by increasing *y* from 0.00 to 0.21, the slope of the PTIs at low partial hydrogen pressures becomes steeper, effectively shifting the PTIs and sensing range to lower pressures. This is in line with the neutron reflectometry results, which show that higher Hf concentrations result in hydrogen being absorbed at lower pressures at 267 °C. Furthermore, there is a clear difference between the PTIs at different temperatures, which can already be seen in Figure 6 but is highlighted in Figure 7. The PTIs of 0.00 ≤ *y* ≤ 0.30 shifts to higher pressures when the temperature is increased, which is expected based on Van ‘t Hoff’s law (eq 1).

**Figure 6:**
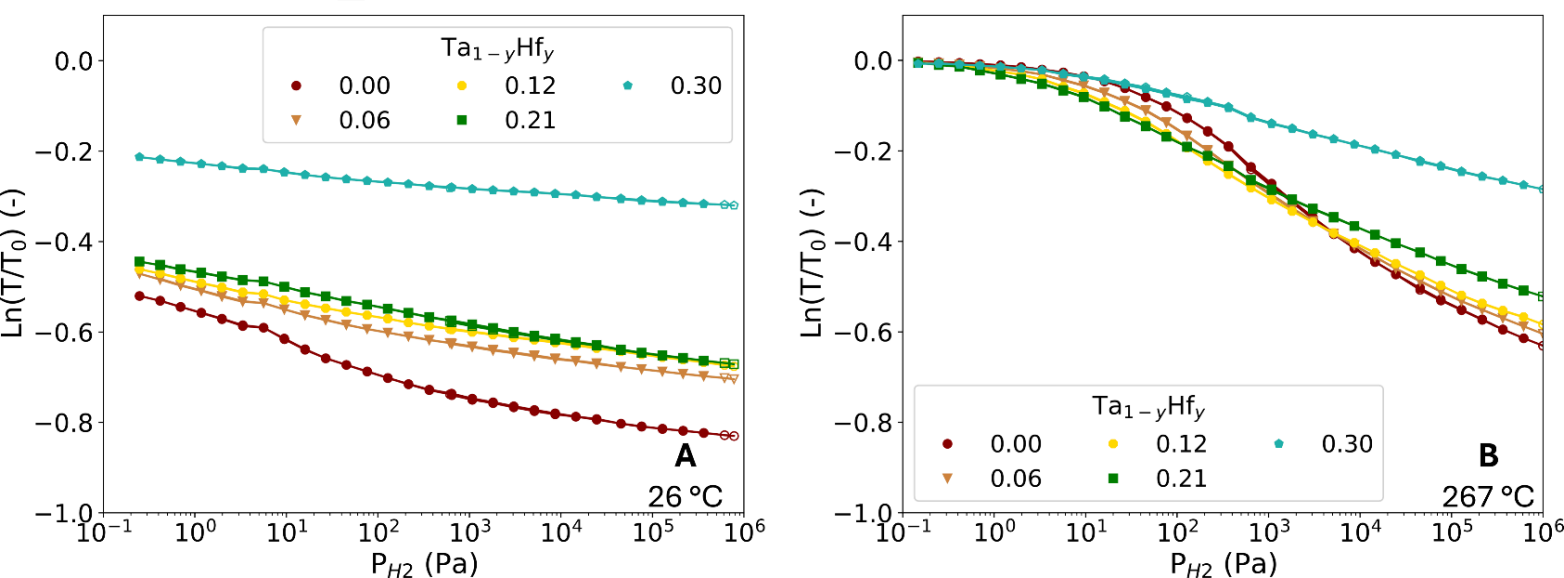
Partial hydrogen pressure dependence of the optical transmission *T* of 40 nm Ta_1–*y*_Hf_*y*_ sensing layer relative to the optical transmission of the unloaded state (*T*_0_) at A) 26 °C and B) 267 °C. The closed markers correspond to increasing pressure steps, and the open markers correspond to decreasing pressure steps.

**Figure 7:**
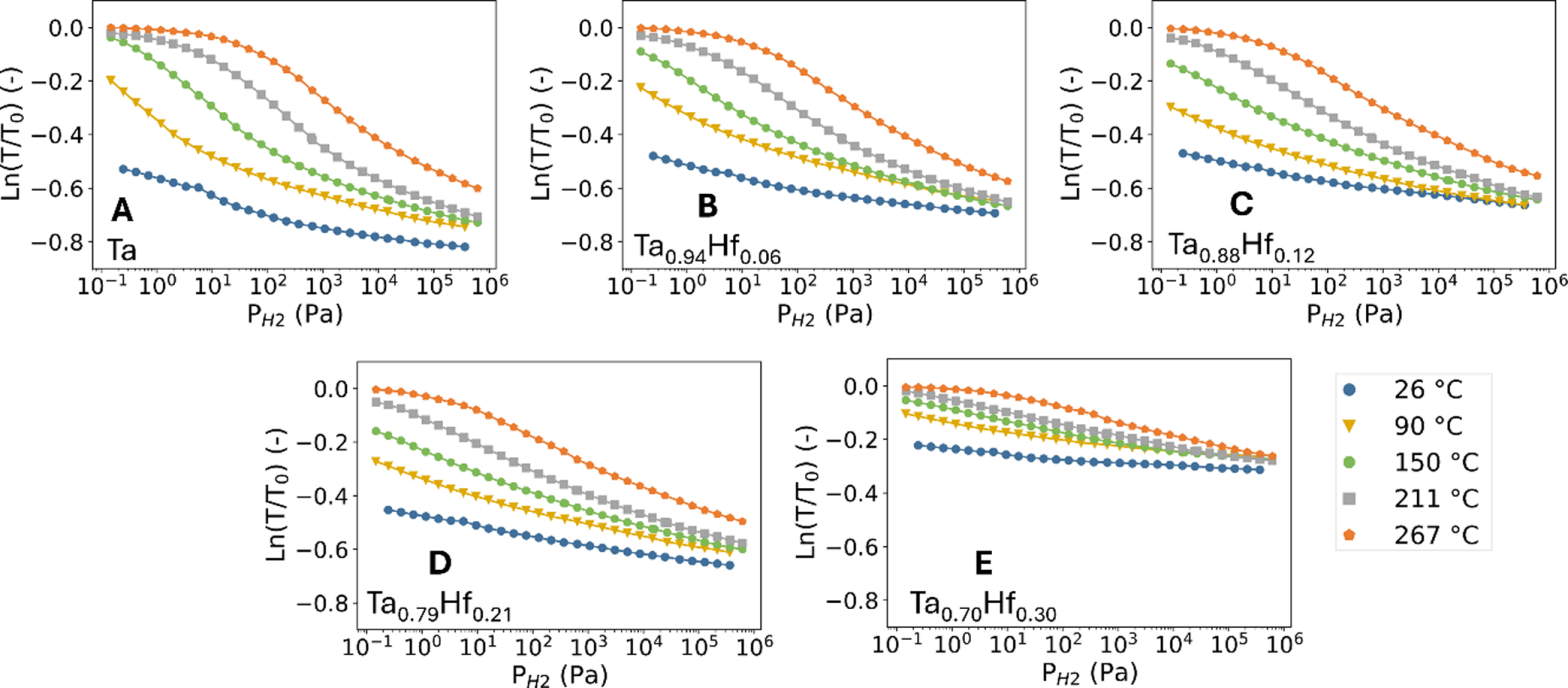
Partial hydrogen pressure and temperature dependence of the optical transmission *T* of the 40 nm Ta_1–*y*_Hf_*y*_ sensing layer relative to the optical transmission of the unloaded state (*T*_0_) of A) Ta, B) Ta_0.94_Hf_0.06_, C) Ta_0.88_Hf_0.12_, D) Ta_0.79_Hf_0.21_, and E) Ta_0.70_Hf_0.30_. Each data point corresponds to the measured optical transmission with decreasing pressure steps.

The long-term stability of the thin films is assessed with an hydrogenation measurement at 267 °C spanning over 140 h, utilizing 10 min cycles. The results of the stability measurement, presented in Figure S11, show that the optical response of the first and last cycle only show small differences, which can be attributed to small deviations of the pressure and temperature. Additionally, AFM micrographs (Figure S3) of the unloaded state show that after exposure the surface is still smooth, and there are no in-plane features. Both the structural and optical measurements show that the Ta_1–*y*_Hf_*y*_ thin films are stable at 267 °C for long periods of time, as no significant changes are observed.

#### 3.2.2 Sensitivity

The sensitivity is a crucial property of hydrogen sensing materials. A highly sensitive sensing material can detect small changes in the partial hydrogen pressure, which is, for example, important for the optimalization of the hydrogen concentration in chemical processes. Here, the sensitivity is defined as the derivative of the average optical response over a partial hydrogen pressure range: 10^–1^ – 10^+2^ Pa, 10^+2^ – 10^+4^ Pa, and 10^+4^ – 10^+6^ Pa, i.e. corresponding to 1.0 to 10^+3^ ppm, 0.1 to 10% and >10% hydrogen. The sensitivities of 0.06 ≤ *y* ≤ 0.30 are normalized with the sensitivity of *y* = 0.00 to show whether the addition of Hf enhances the sensitivity of the sensing material. Figure 8A shows that the sensitivity at 26 °C is reduced with the addition of Hf, especially for lower partial hydrogen pressures. In contrast, the sensitivities of 0.06 ≤ *y* ≤ 0.21 for the lowest partial pressure range (10^–1^ – 10^+2^ Pa) are enhanced significantly compared to Ta when the temperature is increased to 267 °C (Figure 8B). The sensitivity of *y* = 0.30 is reduced compared to that of Ta for each pressure range and all temperatures, which could be related to the likely presence of a secondary phase (see Section 3.1.1).

**Figure 8:**
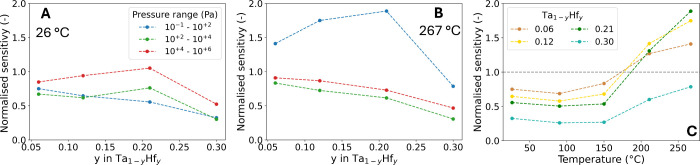
Normalized sensitivity of Ta_0.94_Hf_0.06_, Ta_0.88_Hf_0.12_, Ta_0.79_Hf_0.21_, and Ta_0.70_Hf_0.30_ over multiple pressure ranges at A) 26 °C and B) 267 °C. C) The sensitivity of the 10^–1^ – 10^+2^ Pa pressure range at multiple temperatures. The sensitivity is defined as the average derivative of the optical response of ln­(*T*/*T*_0_) with respect to the partial hydrogen pressure. The sensitivity is normalized with the sensitivity of Ta.

Figure 8C presents the normalized sensitivity of the 10^–1^ – 10^+2^ Pa range per temperature, which shows that the normalized sensitivity increases with the temperature. Furthermore, for 0.06 ≤ *y* ≤ 0.21, the sensitivity becomes greater than the sensitivity of *y* = 0.00 when the temperature is raised to 211 °C by a factor of up to 1.8. Most importantly, the sensitivities of *y* = 0.12 and *y* = 0.21 at 211 and 267 °C for low partial hydrogen pressures are similar to the sensitivity of pure Ta at room temperature. This confirms that the PTIs and the corresponding sensing range of these alloys has shifted enough to reach sufficient sensitivity for the detection of hydrogen at these temperatures. That being said, there is an optimum Hf concentration to obtain the best sensitivity based on the temperature. Figure 8C shows that at 211 °C and at 267 °C, the best sensitivity is obtained by *y* = 0.12 and *y* = 0.21, respectively, while at lower temperatures (25 – 150 °C) the best sensitivity for Ta–Hf alloys is obtained at *y* = 0.06. This shows that the optimal composition is different for each temperature and hydrogen concentration range.

It is important to note that the optical transmission based on the green channel is used for the determination of the sensitivity due to experimental considerations. The wavelength dependence of the optical response is measured, and the results can be found in Figure S12. The results show that the absolute difference between the relative transmission of the thin films when exposed to 0.1% and 100% H_2_, is the largest for wavelengths of 800–900 nm (near-infrared). For 0.00 ≤ *y* ≤ 0.21, this optical contrast between 0.1% and 100% H_2_ is 2.0–3.8 times larger with light of 850 nm compared to 530 nm (most prominent wavelength in the green channel). This shows that the overall sensitivity can be improved by using the near-infrared instead of green light.

#### Decomposition of the Sensitivity

The sensitivity can be decomposed in two different components: the change in the amount of hydrogen absorbed ( $\frac{dx}{dP_{H_{2}}}$ ) and the magnitude by which the optical properties of the material change for every absorbed hydrogen atom ( $d\left(\frac{\ln\left(T/T_{0}\right)}{dx}\right)$ ).^26^ The change in the amount of hydrogen absorbed is determined with neutron reflectometry measurements, of which the PCIs are presented in Figure 4. In this figure, the slope equals $\frac{dx}{dP_{H_{2}}}$ , which is within 5% similar for all compositions at 26 °C. With the similar slopes, it is expected that the sensitivity is also similar. However, as shown in Figure 8A the sensitivity of *y* = 0.21 (and *y* = 0.12) is lower compared to that of Ta for the same pressure range (10^+2^-10^+4^ Pa). This implies that the $d\left(\frac{\ln\left(T/T_{0}\right)}{dx}\right)$ term is reduced with the Hf substitution.

To asses this, Figure 9 presents the absolute value of the optical transmission ln­(*T*/*T*_0_) as a function of the hydrogen concentrations at 26 °C and ∼270 °C. This is done by matching the relation between the partial hydrogen pressure and the hydrogen content *x* (see Figure 4) and the corresponding value of the transmission based on the PTIs found in Figure 6. With Figure 9, we found a linear relationship between the optical response and the hydrogen content. This linear relationship has been reported before in different metal hydrides,^46–48^ but is not trivial, due to the expected changes in the electronic structure in the material with the addition of hydrogen in the interstitial sites.^12,26,48^ Additionally, the measurements at 26 and ∼270 °C follow the same linear trend for each composition. At higher temperature, less hydrogen is absorbed, which in turn decreases the expansion of the unit cell and the optical contrast, but there are no additional thermal effects observed.

**Figure 9:**
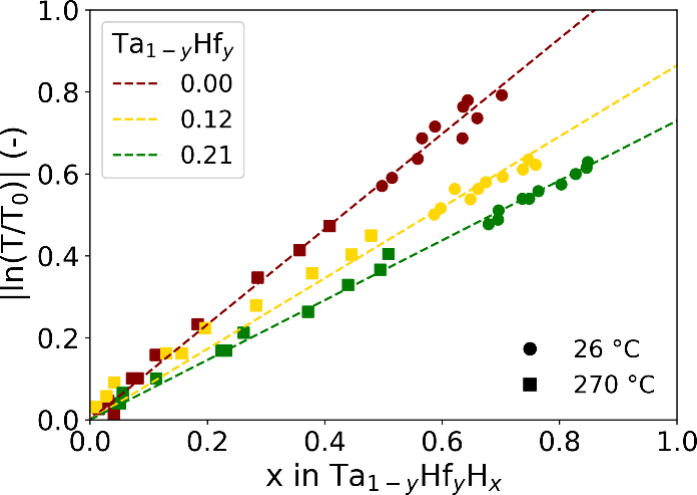
Relation between the hydrogenation and the absolute changes of the optical transmission *T* relative to the unloaded state (*T*_0_) of the Ta_1–*y*_Hf_*y*_ layer at 26 and ∼270 °C. The dotted lines are linear fits constrained to pass through the origin based on the experimental data of both temperatures. The difference in temperature between the neutron reflectometry and hydrogenography measurements is neglected.

Most importantly, the slope $d\left(\frac{\ln\left(T/T_{0}\right)}{dx}\right)$ in Figure 9 decreases significantly with higher Hf concentrations, which negatively impacts the sensitivity. Ideally, we want the slope to be as large as possible to maximize the sensitivity. The decrease in slope results in the lower sensitivity of the Ta–Hf alloys compared to that of Ta. However, this decrease in the $d\left(\frac{\ln\left(T/T_{0}\right)}{dx}\right)$ is offset by an increased value of $\frac{dx}{dP_{H_{2}}}$ at higher temperatures resulting in a higher sensitivity at low partial hydrogen pressure (*P*_H_2__ < 10^+2^ Pa). Additionally, the differences in $d\left(\frac{\ln\left(T/T_{0}\right)}{dx}\right)$ between the compositions explains why the PTIs in Figure 6B intersect, while the PCIs in Figure 4B do not cross each other. While more hydrogen is absorbed, it results in a smaller change in the optical transmission, making it possible for the PTIs to intersect.

#### 3.2.3 Enthalpy and Entropy of Hydrogenation

The shift in the sensing range is largely determined by a change in the hydrogen concentration at a certain pressure. To understand the underlying thermodynamics, we determined the enthalpy and entropy of hydrogenation as a function of the hydrogen concentration using the Van ’t Hoff law (see eq 1) and the PTIs of Figure S13. For this calculation, we selected a set of relative transmission levels, corresponding to a certain hydrogen-to-metal ratio *x*, and determined for each level and temperature the corresponding pressure (*P*_eq_). We may estimate the values of the Δ*H* and Δ*S*, by plotting ln­(*P*_eq_/*P*_0_) as a function of the inverse of the temperature as shown in Figure 10A. The transmission levels are scaled with the relations found in Figure 9 to translate the relative transmission to the corresponding hydrogen content *x*.

**Figure 10:**
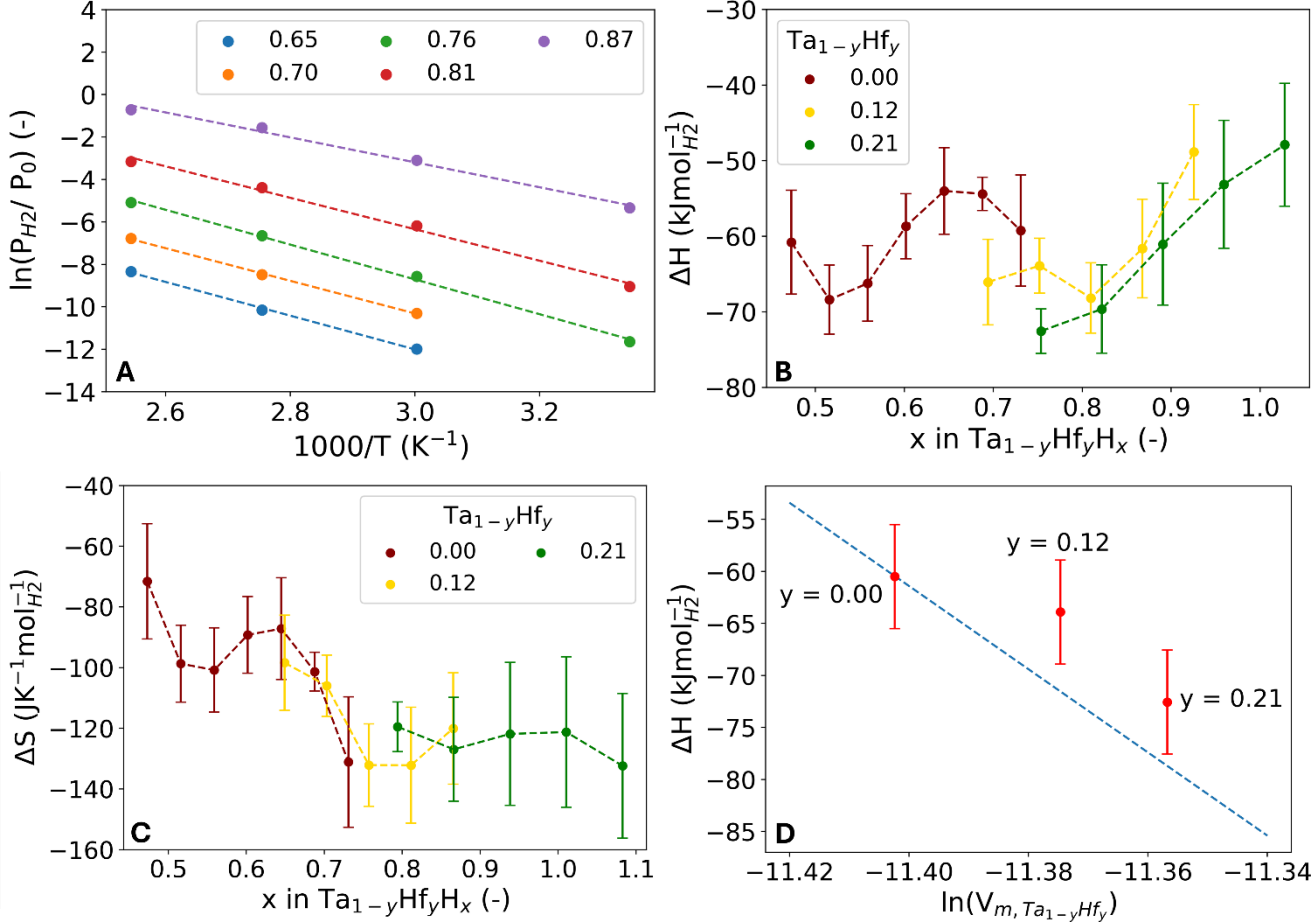
Van ‘t Hoff analysis. A) Fit of Van ’t Hoff’s law (eq 2) to the experimental data for Ta_0.88_Hf_0.12_. The analysis is based on the temperature-dependent optical transmission data of a secondary data set, which can be found in Figure S13. This data set includes measurements at 26, 60, 90, and 120 °C. The optical transmission is converted to the hydrogen content (*x*) that is provided in the figure legend using the relationship found in Figure 9. So, each line corresponds to a fit at a different optical transmission/hydrogen concentration. The B) enthalpy and C) entropy of hydrogenation obtained by the fits of the Van ‘t Hoff law. The error bars represent a 95% confidence interval. D) Relation between the enthalpy of hydrogenation of Ta_1–*y*_Hf_*y*_H_0.75_ and the partial volume of the host metal for a hydrogen concentration of *x* = 0.75. The dashed line is the expected trend based on eq 3, with the bulk modulus of Ta and partial hydrogen concentration in Ta.

The enthalpy values for Ta as shown in Figure 10B, align with the enthalpy bandwidth reported in literature.^35,50–52^ Similar to earlier reports the Δ*H* oscillates with the hydrogen content *x*.^26,51,52^ The results show that by alloying Ta with Hf, the magnitude of Δ*H* increases (becomes more negative) when comparing the same value of *x*. This demonstrates that the original idea of expanding the unit cell to increase the enthalpy is valid. The more favorable enthalpy suggests that a larger amount of hydrogen can be absorbed, which is indeed the case as shown by the neutron reflectometry results in Figure 4. It should be noted that there is little overlap between the *x* – ranges of the different compositions due to the divergence in the amount of hydrogen that is absorbed. Second, the error of the calculated Δ*H* values is quite large. These two factors make it challenging to draw any definitive conclusions.

It is important to note that the relationship between the entropy of hydrogenation and *x* of the different alloy compositions overlaps and follows generally the same trajectory as shown in Figure 10C. Furthermore, the entropy reaches a plateau at a value of Δ*S* = −130 J/(K mol_H2_) for *x* ≥ 0.75, which is the standard entropy of H_2_.^53^ The entropy of hydrogen in a metal consists of both configurational and vibrational contributions. The deviation from the standard entropy at lower hydrogen contents could be related to the configurational contribution. The configurational entropy increases when there are more possible configurations in the system, which is the case when less of the possible sites are occupied by hydrogen.^54^ Moreover, the entropy influences the sensing range of the alloys, as it affects the equilibrium hydrogen pressure for a given temperature (eq 1). So, the shift in sensing range with the Hf-substitution is, among others factors, affected by entropic effects, which may related to the configurational contribution to the entropy as described above.

The enthalpy of hydrogenation of metal hydrides can typically be described as a linear relationship between the molar volume expansion and the bulk modulus. This relation is given by

3 $$\frac{d\Delta H}{d\left(\ln\left(V_{m,Ta_{1-y}Hf_{y}}\right)\right)}=-BV_{m,H_{2}}$$

where *V*_m,Ta_1–*y*_Hf_*y*__ is the molar volume of the host metal, i.e., 1.12 × 10^–5^ m^3^ mol^–1^ for Ta, *B* ≈ 200 GPa is the bulk modulus of Ta and *V*_m,H_2__ = 2 × 10^–6^ m^3^ mol^–1^ is the partial molar volume of hydrogen in the host lattice (based on the XRR layer expansion and corresponding value of *x*).^38^ This suggests that the enthalpy will change proportionally with a certain level of compression or expansion of the material assuming that the bulk modules remains similar. However, the bulk modulus between metals varies, and it is expected that alloying metals affects the bulk modulus. It can be speculated that more compact crystal structures have a larger bulk modulus (lower compressibility) compared to expanded structures, such as Ta–Hf alloys.

The influence of the bulk modulus on the enthalpy is further investigated by using Figure 10D, which presents the relation between the enthalpy of hydrogenation and the partial volume of the host metal. The dashed line is the expected trend of the hydrogenation enthalpy as a function of the Hf content assuming the bulk modulus of Ta to be independent of the Hf content and the partial hydrogen concentration in Ta. Given all uncertainties involving the calculations needed, the enthalpies of *y* = 0.12 and *y* = 0.21 are reasonably consistent with the expected trend. The alignment would strengthen when the bulk modulus decreases as a function of the Hf fraction, which is consistent with the speculation that the Ta–Hf alloys have a smaller bulk modulus compared to Ta. In general, a change in bulk modulus when alloying materials may influence the performance of metals hydride optical hydrogen sensing materials, as it affects the enthalpy of hydrogenation. So, both a change in the bulk modulus and the earlier discussed entropic effects need to be taken into consideration when developing new alloys with the aim to tailor the hydrogen sensing range.

### 3.3 Comparison with Other Sensing Materials

To illustrate the excellent performance at high temperatures (263 ± 5 °C) of the Ta-Hf alloys with respect to earlier researched materials, we compare in Figure 11 the optical response of the best performing alloy, Ta_0.79_Hf_0.21_, with frequently considered hydrogen sensing materials: palladium, palladium–gold, tantalum–ruthenium, and tantalum–palladium. Both Pd and Pd_0.70_Au_0.30_ have no optical response at this temperature and pressure range, making them unsuitable for high-temperature applications. The sensing ranges of Ta_0.94_Ru_0.06_ and Ta_0.94_Pd_0.06_ have both shifted to higher pressures compared to that of Ta, decreasing the sensitivity at lower pressures. This makes Ta_0.94_Ru_0.06_ and Ta_0.94_Pd_0.06_ unsuitable for the detection of leaks at high temperatures. Ultimaly, Ta_0.79_Hf_0.21_ shows the largest sensitivity (slope) at low partial hydrogen pressures at 263 ± 5 °C. This allows the detection of lower partial hydrogen pressures/concentrations and an increased resolution of the sensing material, making it the most promising material in this comparison for high-temperature applications.

**Figure 11:**
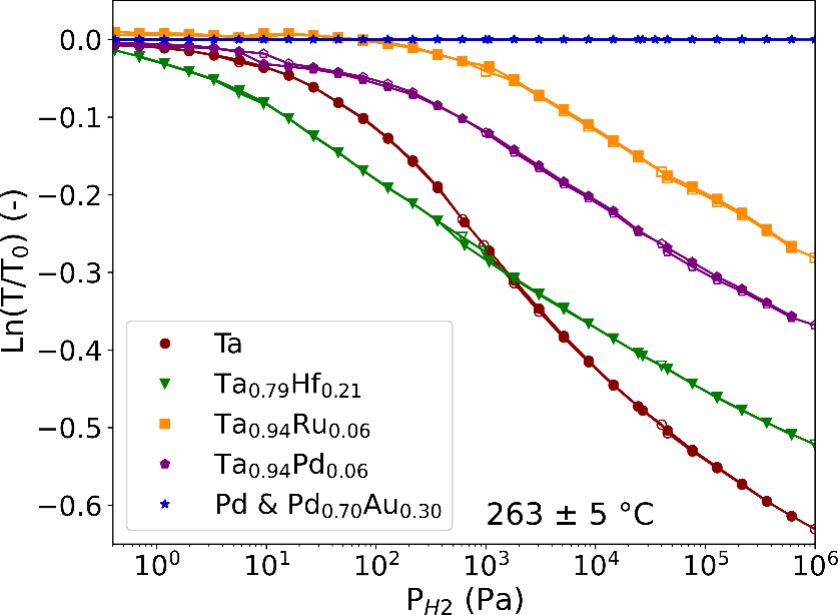
Partial hydrogen pressure dependence of the optical transmission *T* relative to the optical transmission of the unloaded state (*T*_0_) at 263 ± 5 °C of 40 nm Ta_0.79_Hf_0.21_, Ta, Ta_0.94_Ru_0.06_, Ta_0.94_Pd_0.06_, and Pd and Pd_0.70_Au_0.30_ thin films with a 4 nm Ti adhesion layer. The Ta-based thin films also have a 10 nm Pd_0.6_Au_0.4_ capping layer. The closed markers correspond to increasing pressure steps, and the open markers correspond to decreasing pressure steps. The data for the Ta_0.94_Ru_0.06_, Ta_0.94_Pd_0.06_, Pd and Pd_0.70_Au_0.30_ thin films is reprinted with permission from ref 36. Available under a CC-BY 4.0 license. Copyright 2024 Dewi et al.

## 4 Conclusion

In conclusion, we have shown that alloying Ta with the larger element Hf can be used to improve the sensitivity at high temperatures. Ta_1–*y*_Hf_*y*_ thin films with 0.00 ≤ *y* ≤ 0.21 form a solid solution, and alloying Ta with Hf expands the tantalum BCC crystal. In situ XRD and XRR measurements show a gradual expansion of the *d*-spacing and layer thickness of the Ta_1–*y*_Hf_*y*_ thin films upon exposure to hydrogen. In addition, no (first-order) phase transitions or plastic deformations are observed, resulting in a hysteresis-free optical response. Lastly, for 0.00 ≤ *y* ≤ 0.21 the films are structurally stable, and no intermixing with the capping and adhesion layers is observed, not even at 270 °C.

As a result of the Hf alloying, the amount of absorbed hydrogen is increased at room temperature, and at 270 °C a clear shift in the PCI to lower pressures is observed as a function of the Hf concentration. The optical measurements confirm that the shift in PCIs at 270 °C translates into a shift of the sensing range to lower pressures for 0.06 ≤ *y* ≤ 0.21. Moreover, the optical response is hysteresis-free, while the large Ta sensing range spanning over 7 orders of magnitude in partial hydrogen pressure is maintained. The shift in the sensing range results in an improved sensitivity by a factor up to 1.8 for low partial hydrogen pressures compared to Ta for temperatures equal to or above 210 °C. The relation between the shift in sensing range and the expansion of the unit cell is not straightforward, as possible changes in the bulk modulus and entropic effects need to be taken into consideration. Overall, alloys with up to 21% Hf show markedly improved hydrogen sensing properties as compared to Ta for high-temperature applications with an hysteresis-free optical response, large sensing range, excellent stability, and good sensitivity.

## Supplementary Material



## Notes

Section on the analysis of X-ray diffraction (XRD) peak intensity, section and figures on the results of Ta_1–*y*_Hf_*y*_ with 0.40 ≤ *y* ≤ 0.60, table with the sputter conditions for the synthesis of the thin films, figure and table with the fitted parameters of the ex situ X-ray reflection (XRR) measurements of the as-prepared thin films, atomic force microscopy (AFM) surface micrographs of the unloaded thin films, ex situ XRD patterns of the as-prepared thin films, in-plane XRD measurements, in situ XRD and XRR measurements, neutron reflectometry reflectograms and scattering length density (SLD) profiles, long-term optical stability upon cycling, wavelength dependency of the optical transmission, additional hydrogenography measurements (PDF)
