# Supplementary material for: Tantalum–Hafnium: Optical Hydrogen Sensing Materials for High-Temperature Applications
Source: ACS Appl Mater Interfaces. 2025 Jul 18;17(30):43122–34. doi: 10.1021/acsami.5c09600 (PMC12314859; doi:10.1021/acsami.5c09600)
Supplement: Supplementary file 1 [file am5c09600_si_001.pdf]

Supporting information

Tantalum-Hafnium: Optical Hydrogen Sensing Materials for High-Temperature Applications

*Authors: Ilse van Ogtrop [a], Amy Navarathna [a], Herman Schreuders [a], Bernard Dam [a], Lars J. Bannenberg [a]**

[a] Faculty of Applied Sciences, Delft University of Technology, Mekelweg 15, 2629JB Delft, The Netherlands

*Email: l.j.bannenberg@tudelft.nl

Supplementary Texts

Text 1: $Ta_{1-y}Hf_y$ thin films with 0.40 ≤ y ≤ 0.80

Figure S4 and Figure S5 show that $Ta_{1-y}Hf_y$ thin films with y = 0.40 and y = 0.50 are amorphous or microcrystalline as no diffraction peaks are observed. Thins films with y = 0.60 and y = 0.80 do have a BCC structure, but the d-spacing does not follow the linear trend of 0.00 ≤ y ≤ 0.30 (Figure S4C). Nevertheless, the d-spacing of y = 0.60 and y = 0.80 does fall in line with the d_{110}-spacing of BCC Hf. The low intensity of the diffraction peaks of y = 0.60 and y = 0.80 may suggest the presence of an amorphous phase in addition to the BCC structure, similar to y = 0.30. The deviation between the two linear trends of the d_{110}-spacing in Figure S4C may suggest that the BCC structure of y = 0.60 and y = 0.80 is Hf-rich.

Figure S14 shows that the transmission of the $Ta_{1-y}Hf_y$ thin films with 0.40 ≤ y ≤ 0.60 shows an initial change in transmission but almost no change when the thin films are exposed to increasing and decreasing steps of partial hydrogen pressure. The differences in structure could explain the differences in optical response. Similar observations can be made at different temperatures. The PTIs in Figure S15 show the lack of optical response at all temperatures. In addition, the PTIs at 267 °C show instability of the optical response. Overall, the $Ta_{1-y}Hf_y$ thin films with 0.40 ≤ y ≤ 0.60 are unsuitable for optical hydrogen sensing.

Text 2: Intensity XRD peaks

The intensity of the peaks is determined by the summation of the intensity scattered from each individual atom and is depended on the number of electrons of each atom. In theory, as Ta (Z= 73) and Hf (Z=72) have similar amounts of electrons, the intensity of the Ta (110) diffraction peak should remain relatively constant, according to

$$I(hkl) \propto |F(hkl)|^2 = \left|\sum_j f_j \exp\left[2\pi i\left(hx_j + ky_j + lz_j\right)\right]\right|^2 \quad (1)$$

$$f_j \approx Zg(\theta,\lambda)r_e \quad (2)$$

$$I(hkl) \propto Z^2 = \left[Z_{Ta}(1-y) + yZ_{Hf}\right]^2 \quad (3)$$

where $F(hkl)$ is the structure factor, x_j, y_j and z_j the relative coordinates of the atoms in the unit cell and f_j the form factor. The form factor is dependent on the number of electrons, Z, and the diffraction angle in the form of the function $g(\theta,\lambda)$. Important to note, the value of $g(\theta,\lambda)$ decreases when increasing the diffraction angle [1].

References

1. Bannenberg, L. J., Schreuders, H., van Beugen, N., Kinane, C., Hall, S., and Dam, B. (2023) Tuning the Properties of Thin-Film TaRu for Hydrogen-Sensing Applications. *ACS Appl Mater Interfaces*, **15** (6), 8033–8045, doi: 10.1021/acsami.2c20112.

2. Bannenberg, L. J., Blom, L., Sakaki, K., Asano, K., and Schreuders, H. (2023) Completely Elastic Deformation of Hydrogenated Ta Thin Films. *ACS Mater Lett*, **5** (4), 962–969, doi: 10.1021/acsmaterialslett.3c00038.

Supplementary Tables

Table S1: Sputter conditions for the $Ta_{1-y}Hf_y$ thin films. Each film consists of a 4 nm (target thickness) Ti adhesion layer, a 40 nm (target thickness) $Ta_{1-y}Hf_y$ layer and a 10 nm (target thickness) $Pd_{0.6}Au_{0.4}$ capping layer. The Ti layer was sputtered with 100 W for 120 seconds. The capping layer was co-sputtered with Pd (51 W) and Au (27 W) for 52 seconds.

y in $Ta_{1-y}Hf_y$	Power Ta (W)	Power Hf (W)	Time (s)
0.00	130	-	291
0.06	130	12	270
0.12	130	25	249
0.21	130	48	219
0.30	130	77	190
0.40	130	120	160
0.50	130	180	130
0.60	85	177	156
0.80	33	184	193

Table S2: Fitted layer thickness, density, and roughness of the 40 nm $Ta_{1-y}Hf_y$ thin films with a 4 nm Ti adhesion layer and a 10 nm $Pd_{0.6}Au_{0.4}$ capping layer before exposure to hydrogen. The fits are reported in Figure S1. The density of fused quartz was set to 2.64 g/cm^3.

Variable	0	0.06	0.12	0.21	0.30	0.40	0.50	0.60	0.80
$Pd_{0.6}Au_{0.4}$ Thickness (nm)	9.4	9.5	9.6	9.5	9.6	9.5	9.6	9.6	10.4
$Pd_{0.6}Au_{0.4}$ Density (g/cm^3)	14.6	14.5	14.5	14.5	14.8	14.7	14.4	14.5	14.6
$Pd_{0.6}Au_{0.4}$ Roughness (Å)	9.7	9.0	9.3	8.9	9.5	6.9	7.0	7.5	9.1
$Ta_{1-y}Hf_y$ Thickness (nm)	40.3	40.4	40.9	42.3	42.6	42.7	43.3	44.2	46.0
$Ta_{1-y}Hf_y$ Density (g/cm^3)	15.4	15.2	15.0	14.8	14.4	13.7	13.4	13.2	12.8
$Ta_{1-y}Hf_y$ Roughness (Å)	3.0	5.4	6.4	6.4	6.5	8.7	9.3	9.0	9.8
Ti Thickness (nm)	4.5	4.4	4.4	4.1	4.3	4.3	4.3	4.2	4.3
Ti Density (g/cm^3)	4.51	4.51	4.51	4.51	4.51	4.51	4.51	4.51	4.51
Ti Roughness (Å)	5.7	6.2	6.3	6.0	5.6	5.0	6.7	6.2	6.2
Sub. Density (g/cm^3)	2.64	2.64	2.64	2.64	2.64	2.64	2.64	2.64	2.64
Sub. Roughness (Å)	4.2	3.8	4.3	4.6	3.7	3.5	4.5	3.9	4.5

Table S3: SEM-EDS (Jeol, JSM-IT700HR) analysis of mechanically removed material of a $Ta_{0.79}Hf_{0.21}$ thin film (see Figure S2) at a 20,000x magnification. Based on the atom% of Hf and Ta, the sensing layer contains 20.7% Hf and 79.3 % Ta. Error is provided by the JSM-IT700HR analysis software.

Element	*Line*	*Mass%*	*Atom%*
C	*K*	*21.34±0.2*	*70.34±0.7*
O	*K*	*5.10±0.2*	*12.61±0.4*
Ti	*K*	*0.46±0.1*	*0.38±0.1*
Pd	*L*	*5.07±0.2*	*1.89±0.1*
Hf	*M*	*12.31±0.3*	*2.73±0.1*
Ta	*M*	*47.80±0.5*	*10.46±0.1*
Au	*M*	*7.92±0.2*	*1.59±0.1*
Total		*100.00*	*100.00*

Supplementary Figures

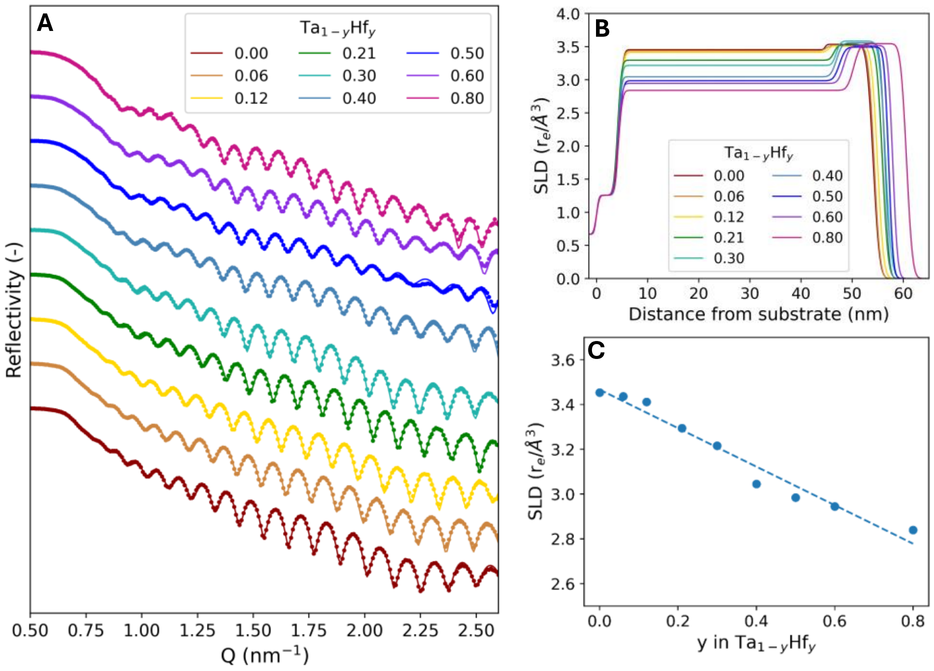

Figure S1: Ex-situ XRR measurements of the as-prepared 40 nm $Ta_{1-y}Hf_y$ thin films with a 4 nm Ti adhesion layer and a 10 nm $Pd_{0.6}Au_{0.4}$ capping layer. A) The continuous lines represent fits of a model to the experimental data presented by the dots. B) SLD profiles of the $Ta_{1-y}Hf_y$ thin films. C) The dependence of the SLD of $Ta_{1-y}Hf_y$ on the hafnium concentration. The dotted line represents the fitted relationship between the SLD and the hafnium concentration.

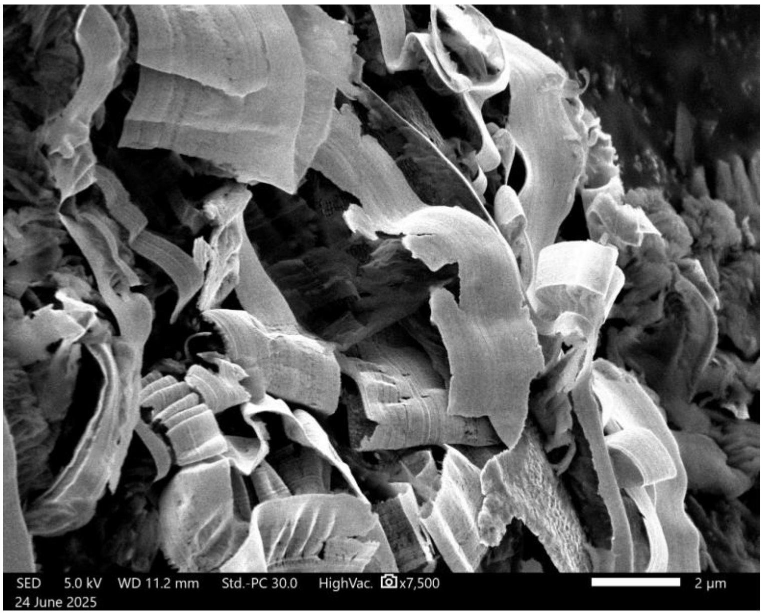

Figure S2: SEM image (Jeol, JSM-IT700HR) of mechanically removed material of a $Ta_{0.79}Hf_{0.21}$ thin film at a 7,500x magnification. Material is removed from the quartz substrate due to the similarities in peak energies between Hf and Si.

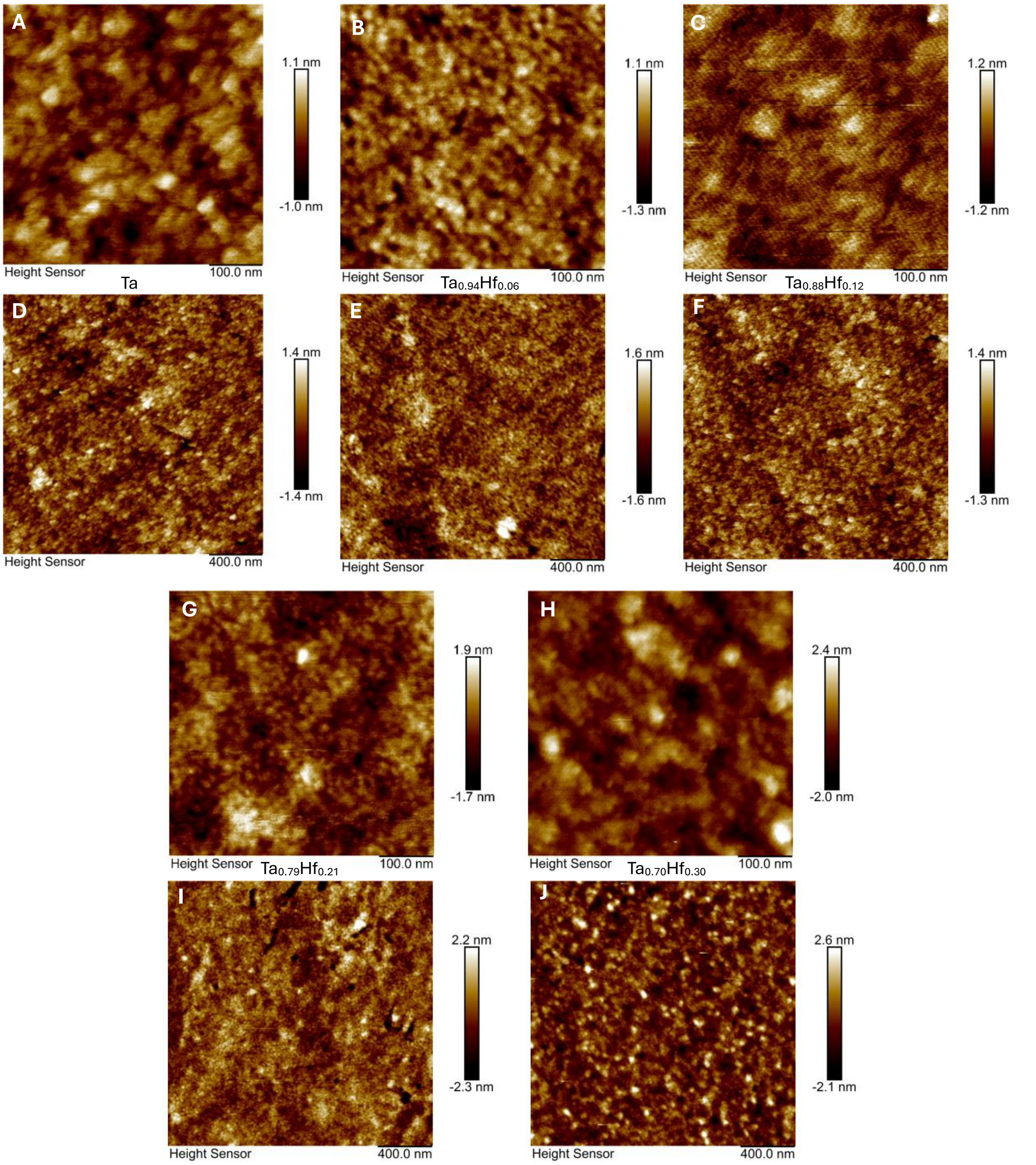

Figure S3: AFM surface micrograph of the unloaded 40 nm A/D) Ta, B/E) $Ta_{0.94}Hf_{0.06}$, D/F) $Ta_{0.88}Hf_{0.12}$, G/I) $Ta_{0.79}Hf_{0.21}$ and H/J) $Ta_{0.70}Hf_{0.30}$ thin films with a 4 nm Ti adhesion layer and a 10 nm $Pd_{0.6}Au_{0.4}$ capping layer over a scan area of A/B/C/G/H) 500 by 500 nm and D/E/F/I/J) 2.0 by 2.0 μm. The AFM scans were made after the thin films were exposed to at least 850 cycles of hydrogen and 270 °C.

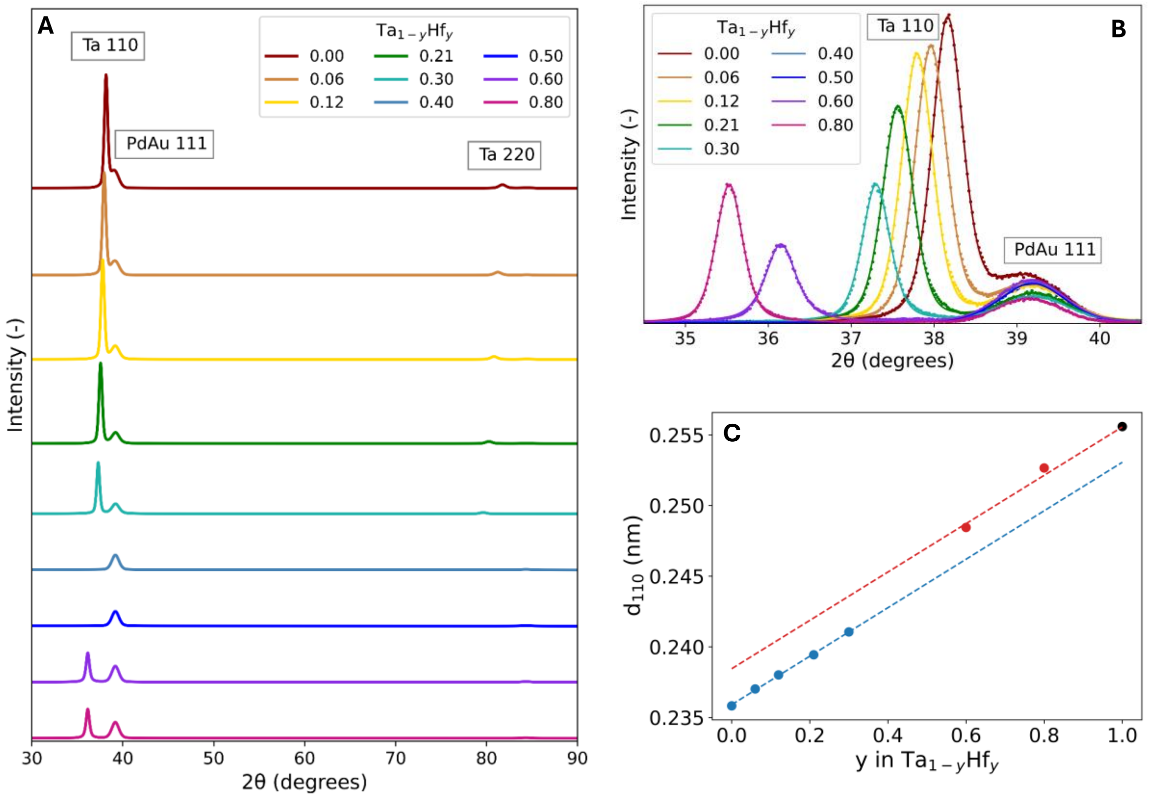

Figure S4: A/B) Fitted ex-situ XRD diffraction patters of the as-prepared 40 nm Ta1-yHfy thin films with a 4 nm Ti adhesion layer and a 10 nm Pd0.6Au0.4 capping layer. The fitting was made with three pseudo-Voight functions and the experimental data. C) The dependence of the d_{110}-spacing in $Ta_{1-y}Hf_y$ on the Hf concentration. The blue fitted line is based on the blue markers of the d_{110}-spacing for $0.00 \leq y \leq 0.30$. The red fitted line is based on the red markers represent the d-spacing of $y = 0.60$ and $y = 0.80$ and the black marker the d_{110}-spacing of a Hf BCC structure.

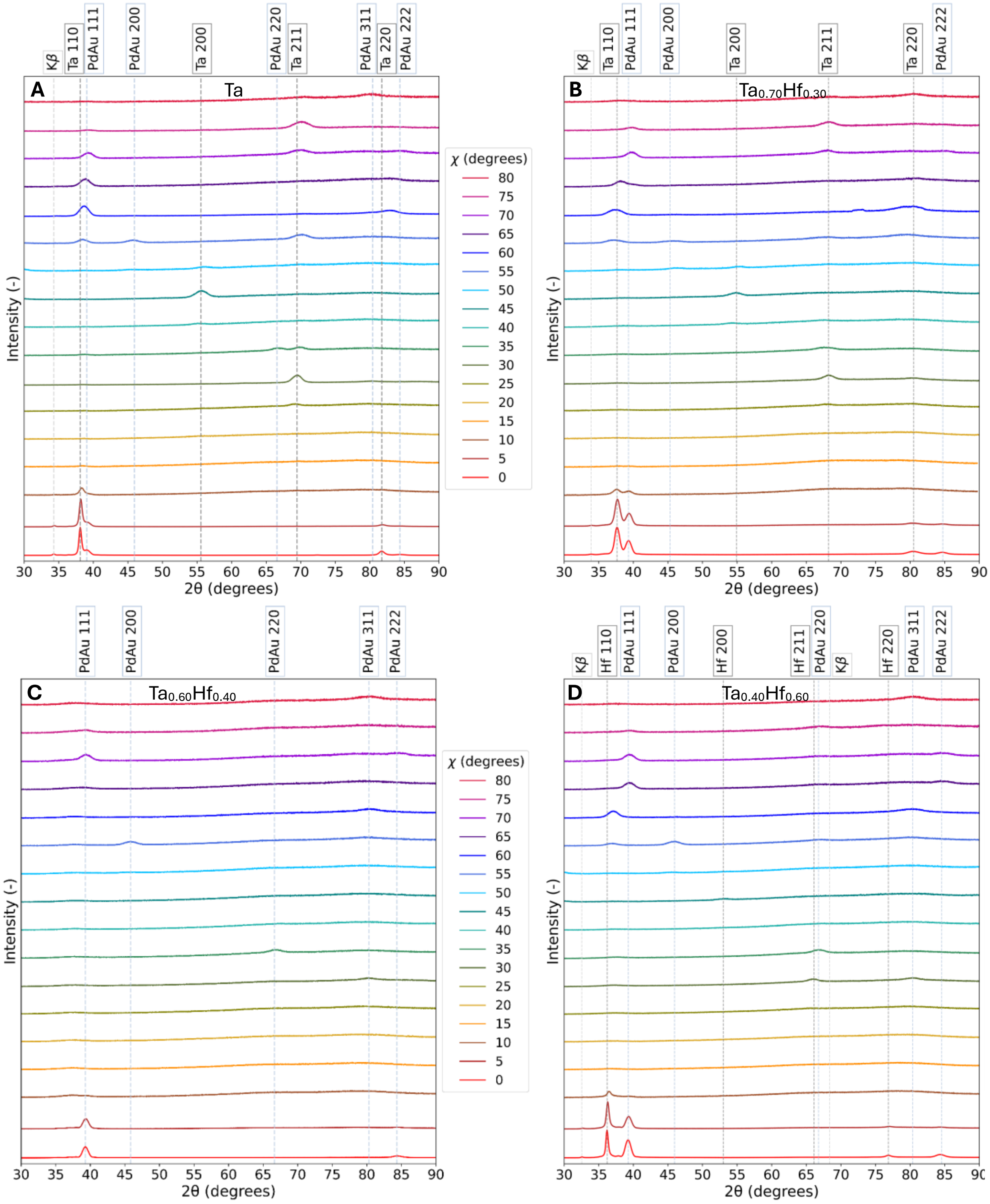

Figure S5: In-plane XRD results of the as-prepared 40 nm A) Ta (representative of 0.00 ≤ y ≤ 0.21), B) $Ta_{0.70}Hf_{0.30}$, C) $Ta_{0.60}Hf_{0.40}$ (representative of y = 0.40 and y = 0.50) and D) $Ta_{0.40}Hf_{0.60}$ (representative of y = 0.60 and y = 0.80) thin film with a 4 nm Ti adhesion layer and a 10 nm $Pd_{0.6}Au_{0.4}$ capping layer measured in air. Each measurement is rotated perpendicular to the direction of the X-ray beam with the value of χ as indicated. The XRD patterns have been normalised to either the maximum intensity (A), B) and C) χ = 0 and 5°) or to a value of 0.4 cps. The dashed lines indicate all the diffraction peaks and are centred on the peak with the lowest χ angle for that peak.

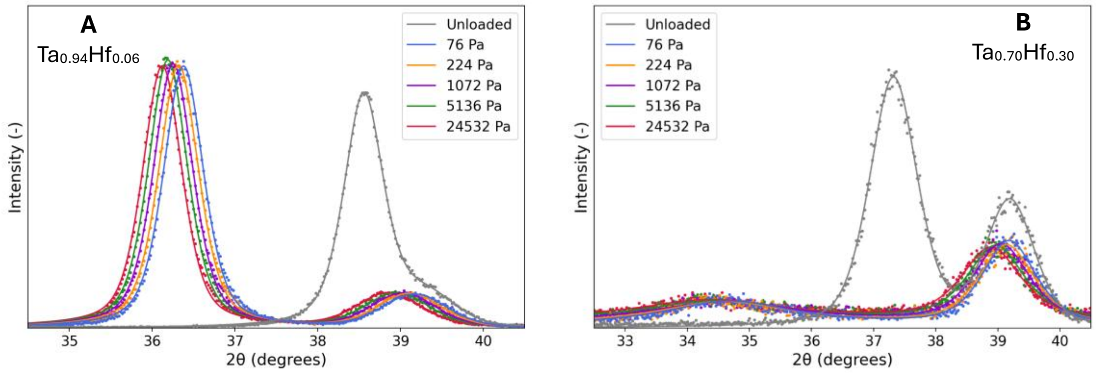

Figure S6: In-situ XRD results of a 40 nm A) $Ta_{0.94}Hf_{0.06}$ and B) $Ta_{0.70}Hf_{0.30}$ thin film with a 4 nm Ti adhesion layer and a 10 nm $Pd_{0.6}Au_{0.4}$ capping layer at 25 °C. The continuous lines represent the fits of two pseudo-Voight functions to the experimental data. Diffraction patterns are measured at the indicated partial H_2 pressures and for decreasing pressure steps.

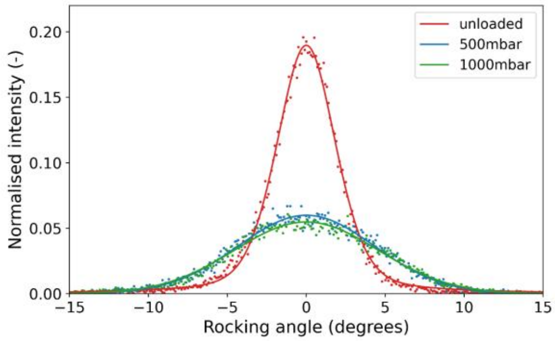

Figure S7: In-situ Rocking curves of the $Ta_{0.79}Hf_{0.21}$ thin film around the Ta (110) peak normalised with the integrated intensity of the experimental data and centred around zero. The continuous lines represent the fits of a pseudo-Voight function to the normalised data represented by the dots.

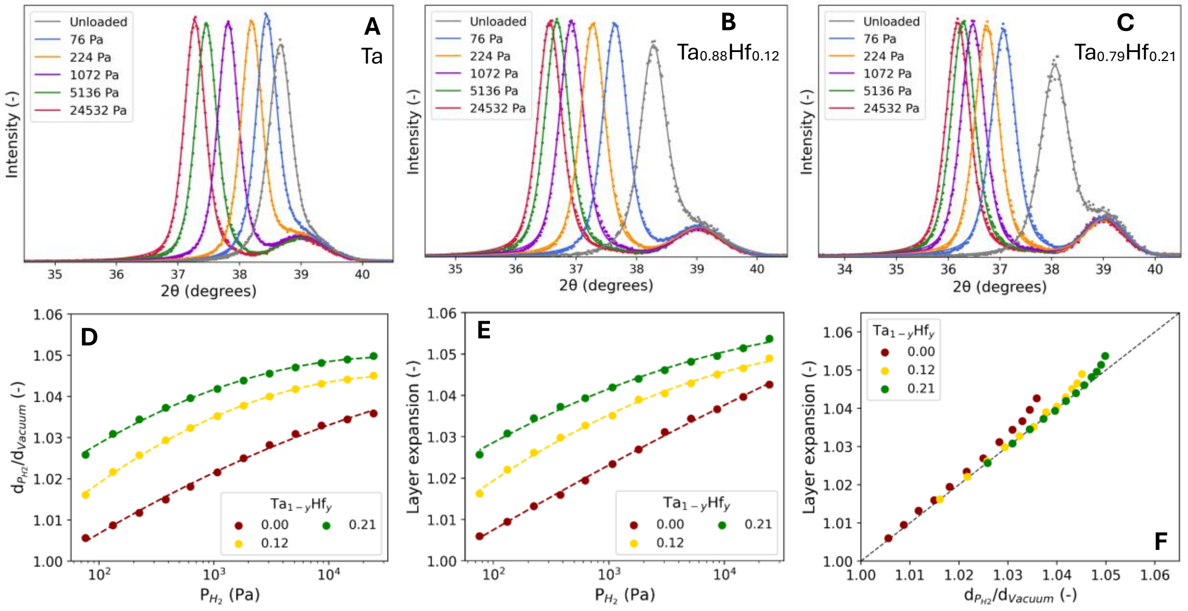

Figure S8: In-situ XRD and XRR results to determine structural changes upon hydrogenation at 270 °C. In-situ XRD results of a 40 nm A) Ta, B) $Ta_{0.88}Hf_{0.12}$ and C) $Ta_{0.79}Hf_{0.21}$ thin film with a 4 nm Ti adhesion layer and a 10 nm $Pd_{0.6}Au_{0.4}$ capping layer at 270 °C. Diffraction patterns are measured at the indicated partial hydrogen pressures and for decreasing pressure steps. The continuous lines represent the fits of two pseudo-Voight functions to the experimental data. D) Partial hydrogen pressure dependence of the d_{110}-spacing expansion relative to the unloaded state, d_{Vavuum}, measured at 270 °C in vacuum. The dotted lines represent the fits of a second-degree polynomial function to the experimental data. E) The dependence of the $Ta_{1\text{-}y}Hf_y$ layer expansion on the partial hydrogen pressure based on the in-situ XRR results. The dashed lines represent fits of a second-degree polynomial function to the experimental data presented by the dots. F) Relation between the d_{110}-spacing and layer expansion. The black dashed line represents the $V \propto d_{hkl}$ relationship.

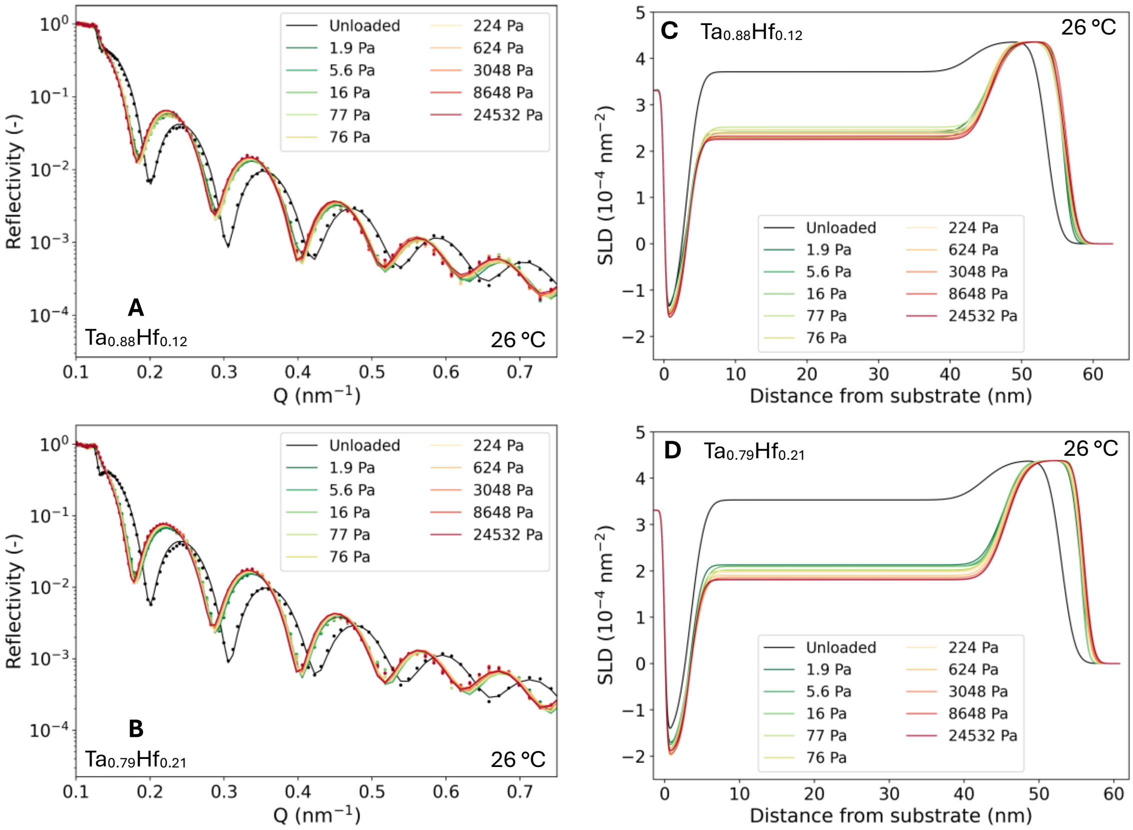

Figure S9: In-situ neutron reflectometry results of the 40 nm $Ta_{1-y}Hf_y$ thin films with a 4 nm Ti adhesion layer and a 10 nm $Pd_{0.6}Au_{0.4}$ capping layer at 26 °C. A-B) Reflectograms of the $Ta_{1-y}Hf_y$ thin films measured for the hydrogen pressures indicated in the legend and for decreasing pressure steps. The continuous lines represent fits of a 3-layer model to the data. The fits provide estimates of the scattering length density and layer thickness to calculate the hydrogen content x in the thin films ($Ta_{1-y}Hf_yH_x$) following Eq. 2. C-D) Scattering length density (SLD) profiles. The in-situ neutron reflectometry results for the Ta thin film at 25 °C can be found in the SI of [2].

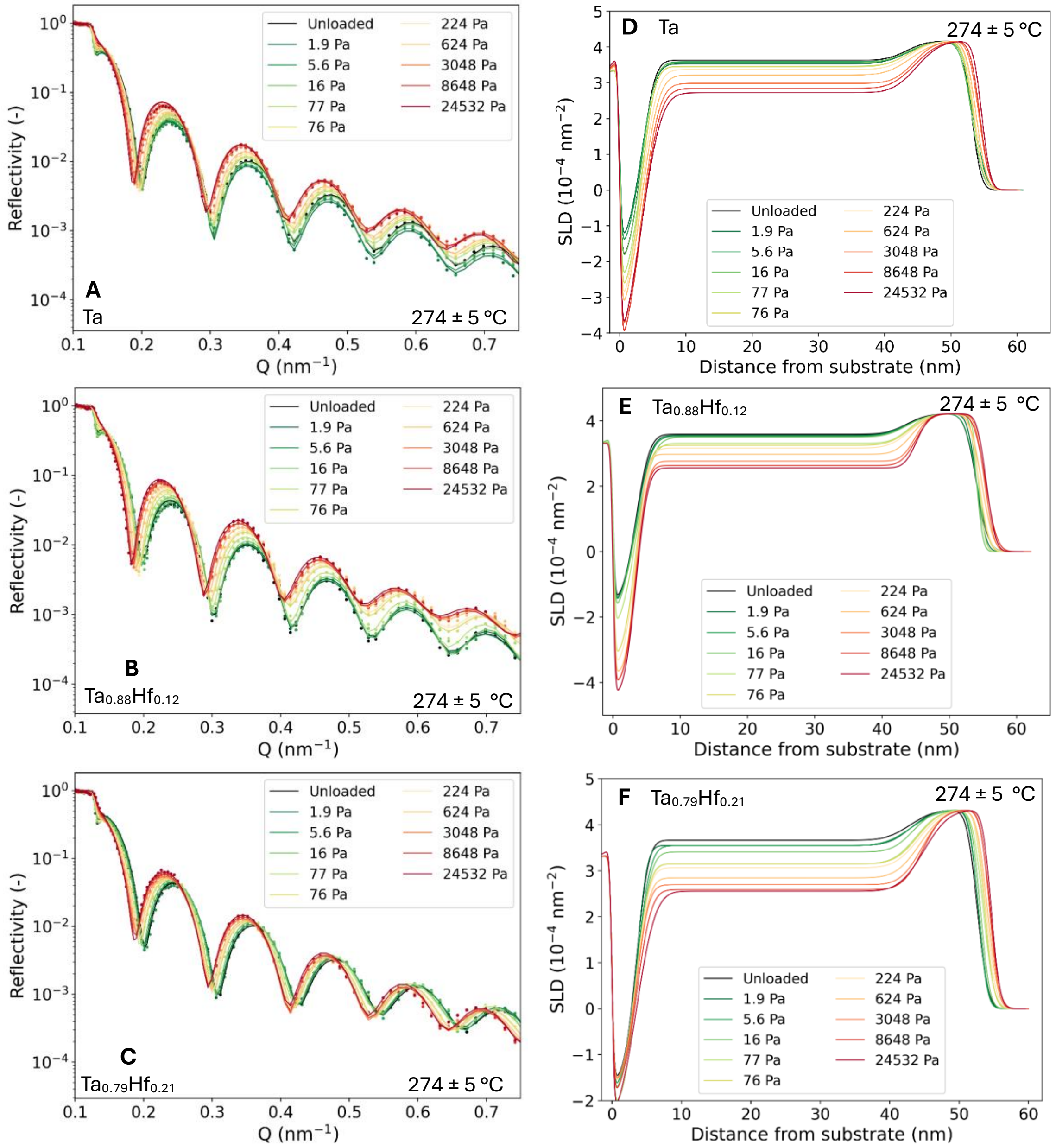

Figure S10: In-situ neutron reflectometry results of the 40 nm $Ta_{1-y}Hf_y$ thin films with a 4 nm Ti adhesion layer and a 10 nm $Pd_{0.6}Au_{0.4}$ capping layer at 274 ± 5 °C. A-C) Reflectograms of the $Ta_{1-y}Hf_y$ thin films measured for the hydrogen pressures indicated in the legend and for decreasing pressure steps. The continuous lines represent fits of a 3-layer model to the data. The fits provide estimates of the scattering length density and layer thickness to calculate the hydrogen content x in the thin films ($Ta_{1-y}Hf_yH_x$) following Eq. 2. E-F) Scattering length density (SLD) profiles.

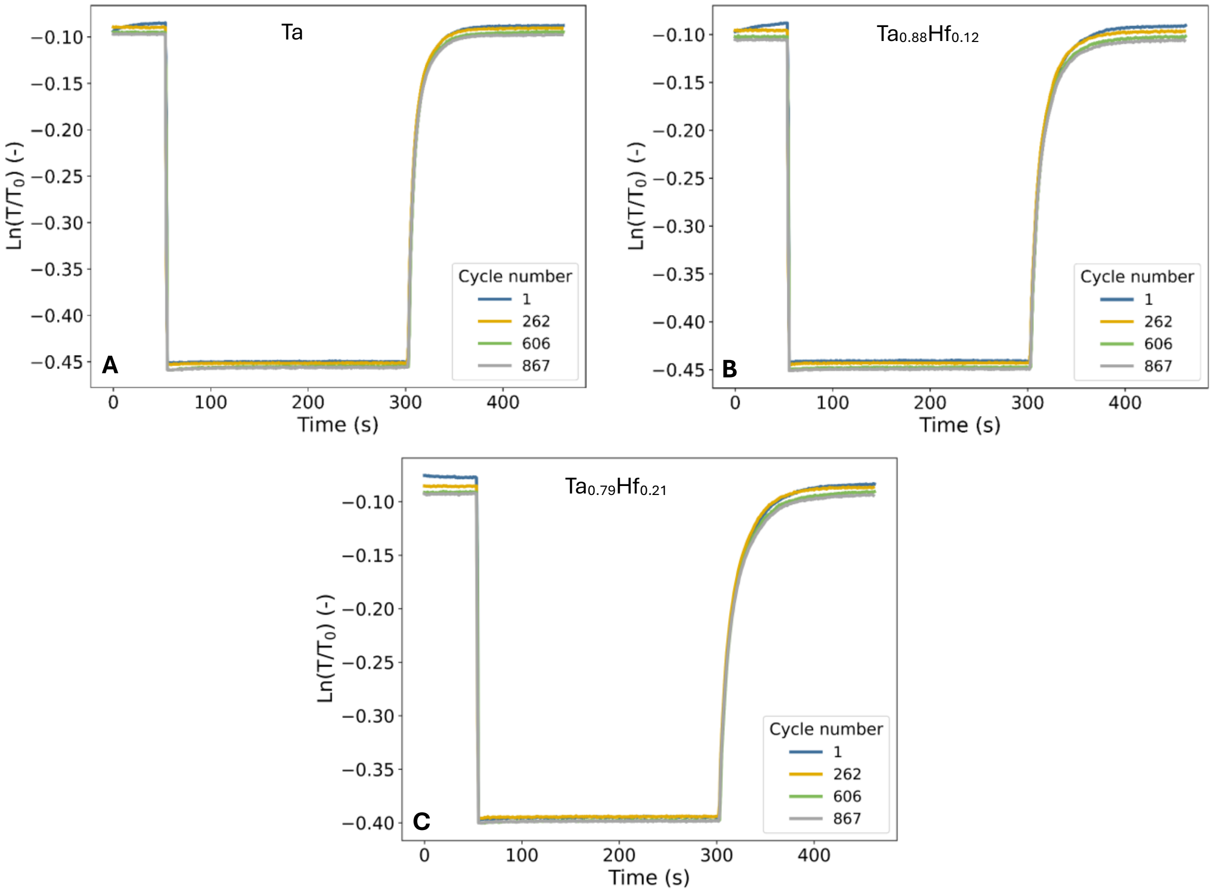

Figure S11: Stability of the 40 nm A) Ta, B) $Ta_{0.88}Hf_{0.12}$, and C) $Ta_{0.79}Hf_{0.12}$ thin films with a 4 nm Ti adhesion layer and a 10 nm $Pd_{0.6}Au_{0.4}$ capping layer at 267 °C. The pressure was varied between partial hydrogenation (P_{H2} = 4.0 · 10^{+3} Pa) and partial dehydrogenation (P_{H2} = 10 Pa) for ~850 cycles.

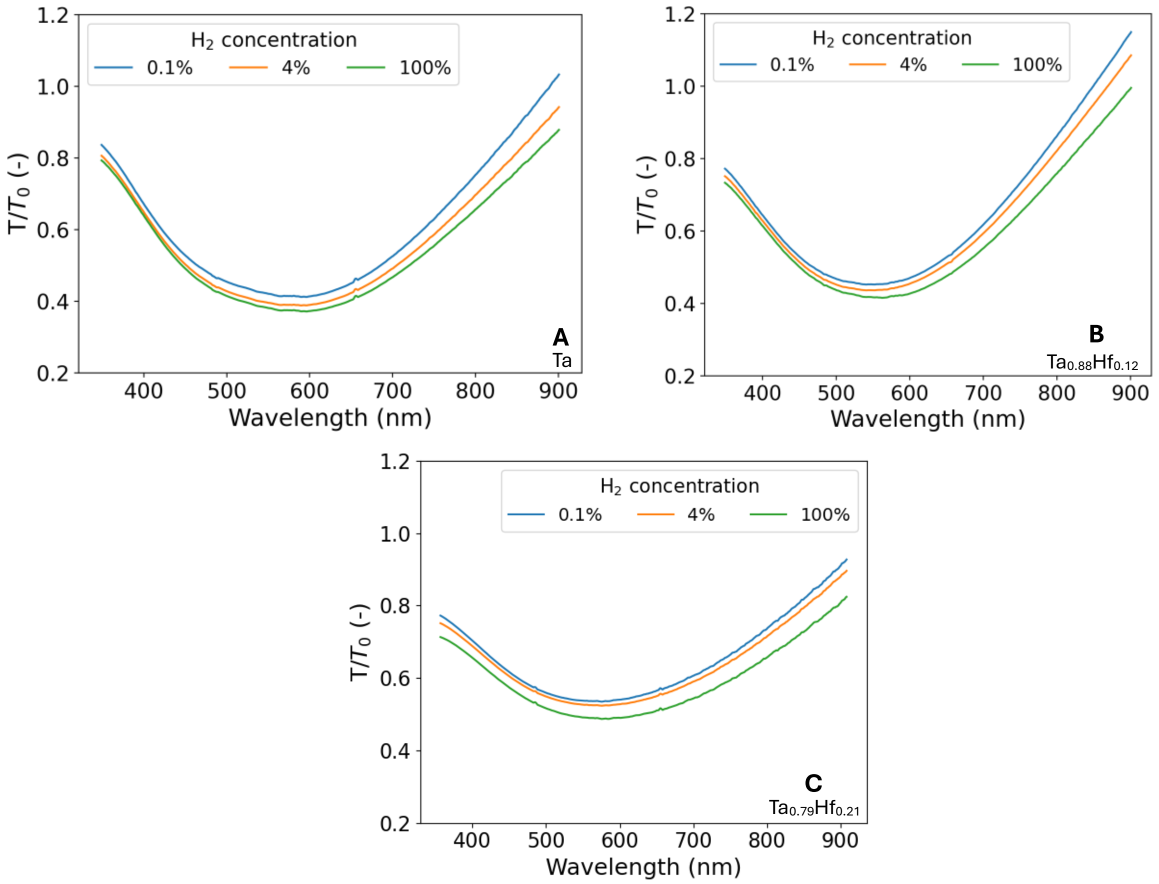

Figure S12: Wavelength dependence of optical transmission T relative to the transmission of the unloaded state T_0 of the 40 nm A) Ta, B) $Ta_{0.88}Hf_{0.12}$ and C) $Ta_{0.79}Hf_{0.21}$ thin films with a 4 nm Ti adhesion layer and a 10 nm $Pd_{0.6}Au_{0.4}$ capping layer at room temperature.

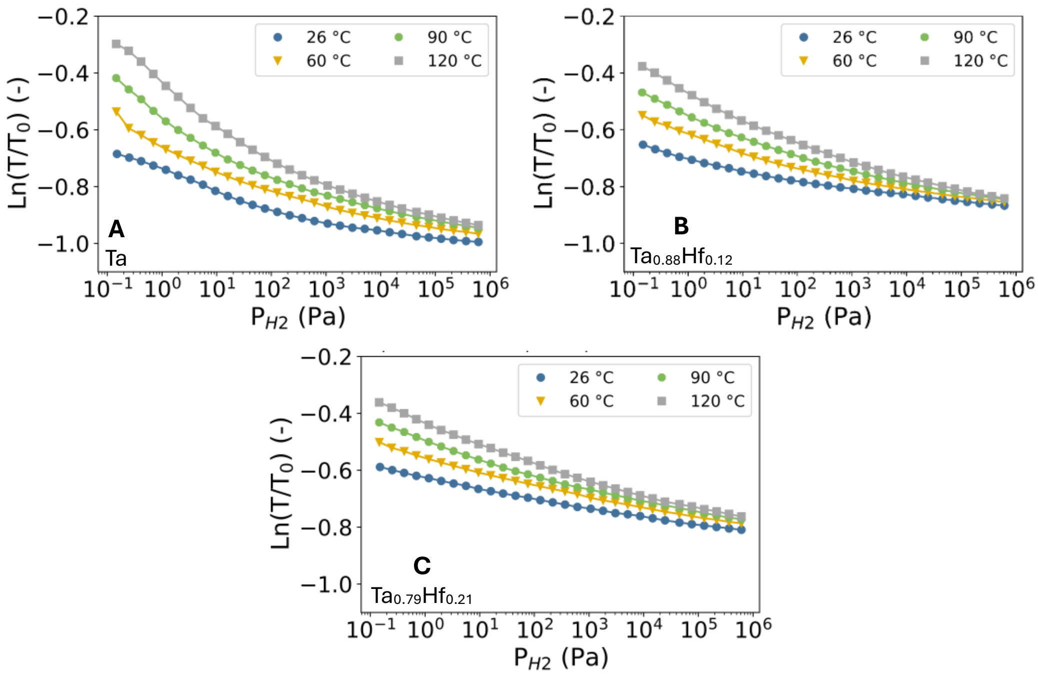

Figure S13: Partial hydrogen pressure and temperature dependence of the optical transmission T of 40 nm $Ta_{1-y}Hf_y$ sensing layer relative to the optical transmission of the unloaded state (T_0) of A) Ta, B) $Ta_{0.88}Hf_{0.12}$, and C) $Ta_{0.79}Hf_{0.21}$. Each data point corresponds to the measured optical transmission with decreasing pressure steps.

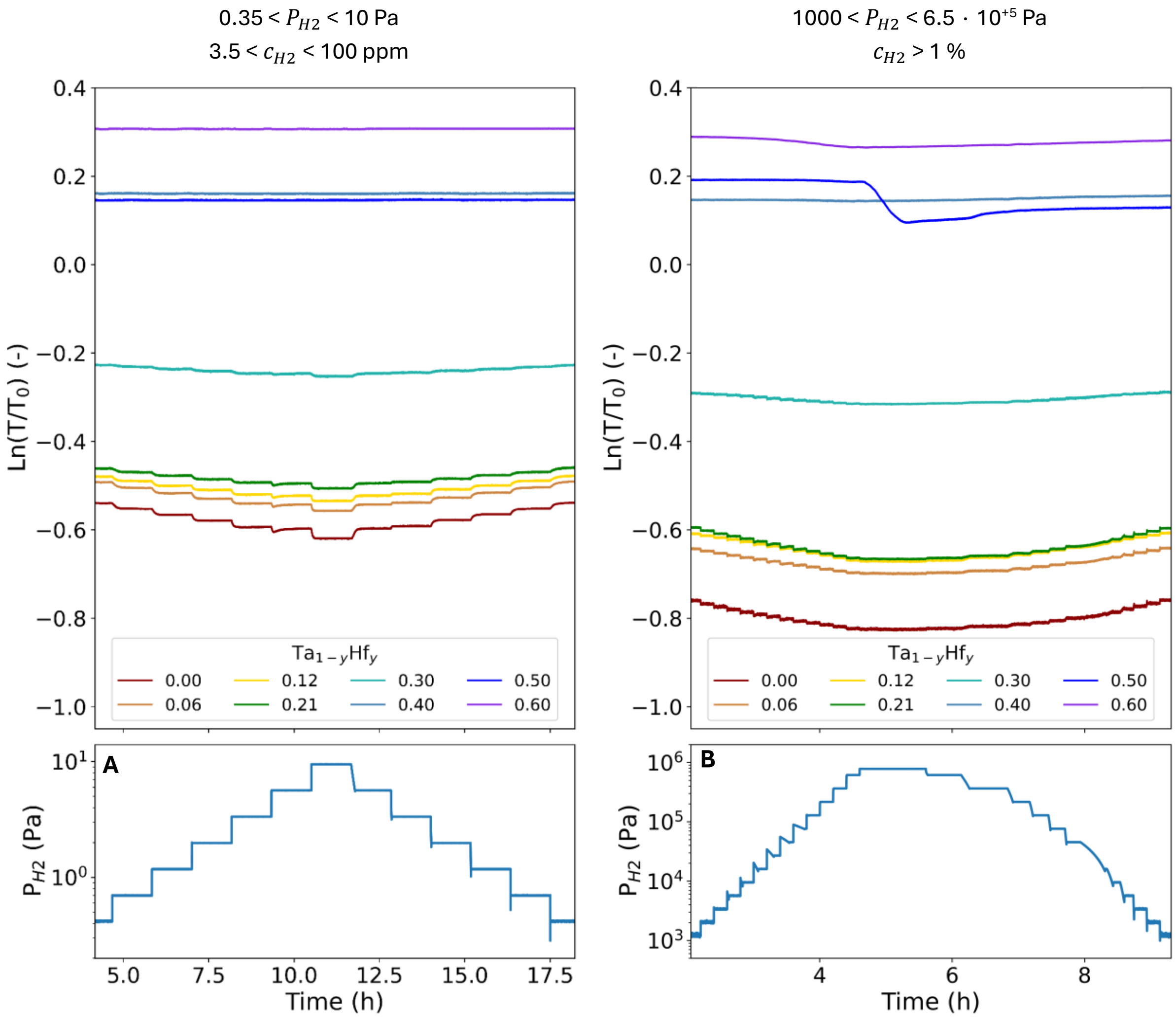

Figure S14: Changes of the optical transmission T relative to the transmission of the unloaded state T_0 of the 40 nm $Ta_{1-y}Hf_y$ thin films with a 4 nm Ti adhesion layer and a 10 nm $Pd_{0.6}Au_{0.4}$ capping layer. The contribution of the capping layer was subtracted by subtracting the optical response of the $Ta_{0.5}Au_{0.5}$ reference thin film with the same adhesion and capping layer. The thin films were exposed at 25 °C to various increasing and decreasing pressure steps of A) $1.5 \cdot 10^{-1} < P_{H2} < 1.0 \cdot 10^{+1}$ Pa and B) $1.0 \cdot 10^{+3} < P_{H2} < 6.5 \cdot 10^{+5}$ Pa. The indicated hydrogen concentration ranges are determined for an environment with a total pressure of 10^{+5} Pa.

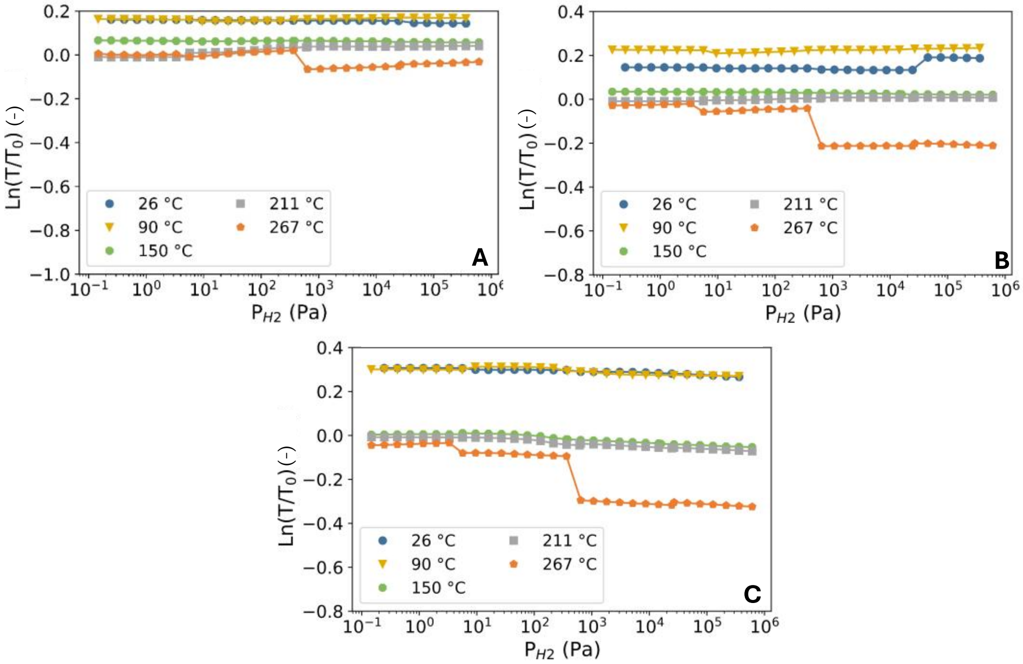

Figure S15: Partial hydrogen pressure and temperature dependence of the optical transmission T of 40 nm $Ta_{1-y}Hf_y$ sensing layer relative to the optical transmission of the reference sample of A) $Ta_{0.60}Hf_{0.40}$, B) $Ta_{0.50}Hf_{0.50}$, and C) $Ta_{0.40}Hf_{0.60}$. Each data point corresponds to the measured optical transmission with decreasing pressure steps.
